# Changes in correlation between promoter methylation and gene expression in cancer

**DOI:** 10.1186/s12864-015-1994-2

**Published:** 2015-10-28

**Authors:** Matahi Moarii, Valentina Boeva, Jean-Philippe Vert, Fabien Reyal

**Affiliations:** CBIO-Centre for Computational Biology, Mines Paristech, PSL-Research University, 35 Rue Saint-Honore, Fontainebleau, F-77300 France; Department of Bioinformatics, Biostatistics and System Biology, Institut Curie, 11-13 Rue Pierre et Marie Curie, Paris, F-75248 France; U900, INSERM, 11-13 Rue Pierre et Marie Curie, Paris, F-75248 France; UMR932, Immunity and Cancer Team, Institut Curie, 26 Rue d’Ulm, Paris, 75006 France; Department of Translational Research, Residual Tumor and Response to Treatment Team, Institut Curie, 26 Rue d’Ulm, Paris, 75006 France; Department of Surgery, Institut Curie, 26 Rue d’Ulm, Paris, 75006 France

**Keywords:** Epigenetic regulation, Cancer, CpG Island methylator phenotype

## Abstract

**Background:**

Methylation of high-density CpG regions known as CpG Islands (CGIs) has been widely described as a mechanism associated with gene expression regulation. Aberrant promoter methylation is considered a hallmark of cancer involved in silencing of tumor suppressor genes and activation of oncogenes. However, recent studies have also challenged the simple model of gene expression control by promoter methylation in cancer, and the precise mechanism of and role played by changes in DNA methylation in carcinogenesis remains elusive.

**Results:**

Using a large dataset of 672 matched cancerous and healthy methylomes, gene expression, and copy number profiles accross 3 types of tissues from The Cancer Genome Atlas (TCGA), we perform a detailed meta-analysis to clarify the interplay between promoter methylation and gene expression in normal and cancer samples. On the one hand, we recover the existence of a CpG island methylator phenotype (CIMP) with prognostic value in a subset of breast, colon and lung cancer samples, where a common subset of promoter CGIs hypomethylated in normal samples become hypermethylated. However, this hypermethylation is not accompanied by a decrease in expression of the corresponding genes, which are already lowly expressed in the normal genes. On the other hand, we identify tissue-specific sets of genes, different between normal and cancer samples, whose inter-individual variation in expression is significantly correlated with the variation in methylation of the 3’ flanking regions of the promoter CGIs. These subsets of genes are not the same in the different tissues, nor between normal and cancerous samples, but transcription factors are over-represented in all subsets.

**Conclusion:**

Our results suggest that epigenetic reprogramming in cancer does not contribute to cancer development via direct inhibition of gene expression through promoter hypermethylation. It may instead modify how the expression of a few specific genes, particularly transcription factors, are associated with DNA methylation variations in a tissue-dependent manner.

**Electronic supplementary material:**

The online version of this article (doi:10.1186/s12864-015-1994-2) contains supplementary material, which is available to authorized users.

## Background

DNA methylation is one of the main epigenetic mechanisms, alongside histone modifications, that plays a significant role in gene silencing [1], tissue differentiation [2], cellular development [3], X-chromosome inactivation [4], or genetic imprinting [5]. Aberrant hyper-methylation of high-density CpG regions known as CpG Islands (CGIs) [6] and genome-wide hypo-methylation [7] have often been associated with cancer and there has been an increasing effort to understand the specific epigenetic modifications that contribute to carcinogenesis [8–10]. In addition to promoter CGIs themselves, their surrounding area called shores (up to 2kb from CGIs) and shores (2kb to 4kb from CGIs) have also a cancer- and tissue-specific methylation [11], while even larger cancer-specific methylation variations were reported in so called open sea regions, far from CGIs [12]. In this study, we focus on methylation in promoter CGIs and surrounding regions only, in order to investigate its association in cis with gene expression.

The possibility to quantify DNA methylation genome-wide on normal and cancer tissues, with microarray or sequencing technologies, has triggered a lot of data-driven research to clarify the role of methylation in gene regulation and cancer. Several studies have highlighted a correlation between differentially methylated regions near promoter regions and gene expression changes [13–17]. However, it has also been reported that aberrant over-methylation occurs mostly in normally down-regulated genes, questioning the role of methylation as a causal mechanism for gene repression [18–21]. More recently, Timp et al. have proposed a model where epigenetic aberrations contribute to carcinogenesis by dysregulating the functions of specific genes that regulate the epigenome itself [22, 23]. Reddington et al. speculate that epigenetic reprogramming might lead to an altered Polycomb binding landscape which could impact genome regulation [24].

To gain further insight into the role of DNA methylation in cancer, we perform a large-scale meta-analysis of methylation profiles of normal and cancerous samples from multiple tissues from The Cancer Genome Atlas (TCGA). For each CGI and surrounding area, we focus on (i) its average methylation profile and (ii) the association between variations of its methylation profile and variations in the expression of the target gene. Comparing these parameters between normal and cancerous samples of different tissues suggests that the interplay between promoter methylation and gene expression, and how they are modified in cancer, is not simple. On the one hand, while each promoter CGI tends to be either hypo- or hypermethylated in all normal samples, we observe hypermethylation of a common subset of CGIs in several cancer samples of different tissues (breast, lung and colon), supporting the existence of a CpG island methylator phenotype (CIMP) with prognostic value, as introduced by Toyota et al. [25]. However, we did not find evidence that the genes associated with hypermethylated promoter CGIs were less expressed in the cancer samples, as most of the genes concerned are already lowly expressed in normal tissues, as already observed by [19]. On the other hand, looking more precisely at associations between promoter methylation level and gene expression within a set of samples, we observe for each tissue and each normal or cancerous sample set a subset of genes, different from the genes hypermethylated in the CIMP phenotype, for which this association is important and strongest outside of the CGIs, namely in the N-shores and N-shelves. This subset of genes varies across tissues but also whether we consider healthy or cancerous samples. However, transcription factors are over-represented in all subsets. This suggests that epigenetic reprogramming might contribute to carcinogenesis in part by modifying gene expression susceptibility to changes in DNA methylation.

## Results

### Classification of genes based on their CGI methylation profiles in normal and cancerous tissues

We first assess how promoter methylation profiles differ between genes, when for each gene we consider the average methylation profile across normal or cancerous samples. For that purpose, we collected high-density methylation datasets from the cancer genome atlas (TCGA) data portal providing more than 485K CpG methylation levels for 672 normal and cancerous samples from three tissues of origin: breast, colon and lung (Table 1). For each CGI, we combine the probes in the CGI and in the shore and shelves of the CGI, defined as the regions up to 4kb outside of the CGI [11], in a unique CGI, shores and shelves (CGI + SS) methylation profile. We restrict our analysis to the 1827 CGI + SS where at least 20 CpG probes are measured by the technology in order to have high enough coverage to measure the methylation variation within each CGI + SS. For each of the three tissue of origin, and each normal or cancerous set of tissues, we compute the average methylation profile of each CGI + SS by averaging the methylation values of each CpG across the samples. Hence we compute 3×2=6 average profile for each CGI + SS, with we refer to below as *CGI+SS signatures*.

**Table 1:** Patients dataset. Original dataset sizes for methylation (Meth), gene expression (GE) and CNV profiles for normal (N) or cancerous (C) tissues. The “Matched” column represents the final dataset containing samples with matched methylation, gene expression and copy number profiles

	Meth		GE		CNV		Matched	
	N	C	N	C	N	C	N	C
Breast	97	626	100	778	1073	1041	70	474
Colon	38	291	0	193	0	470	0	33
Lung	32	452	37	125	568	516	13	82
Total	167	1370	137	1096	1641	1981	83	589

**Fig. 1:**
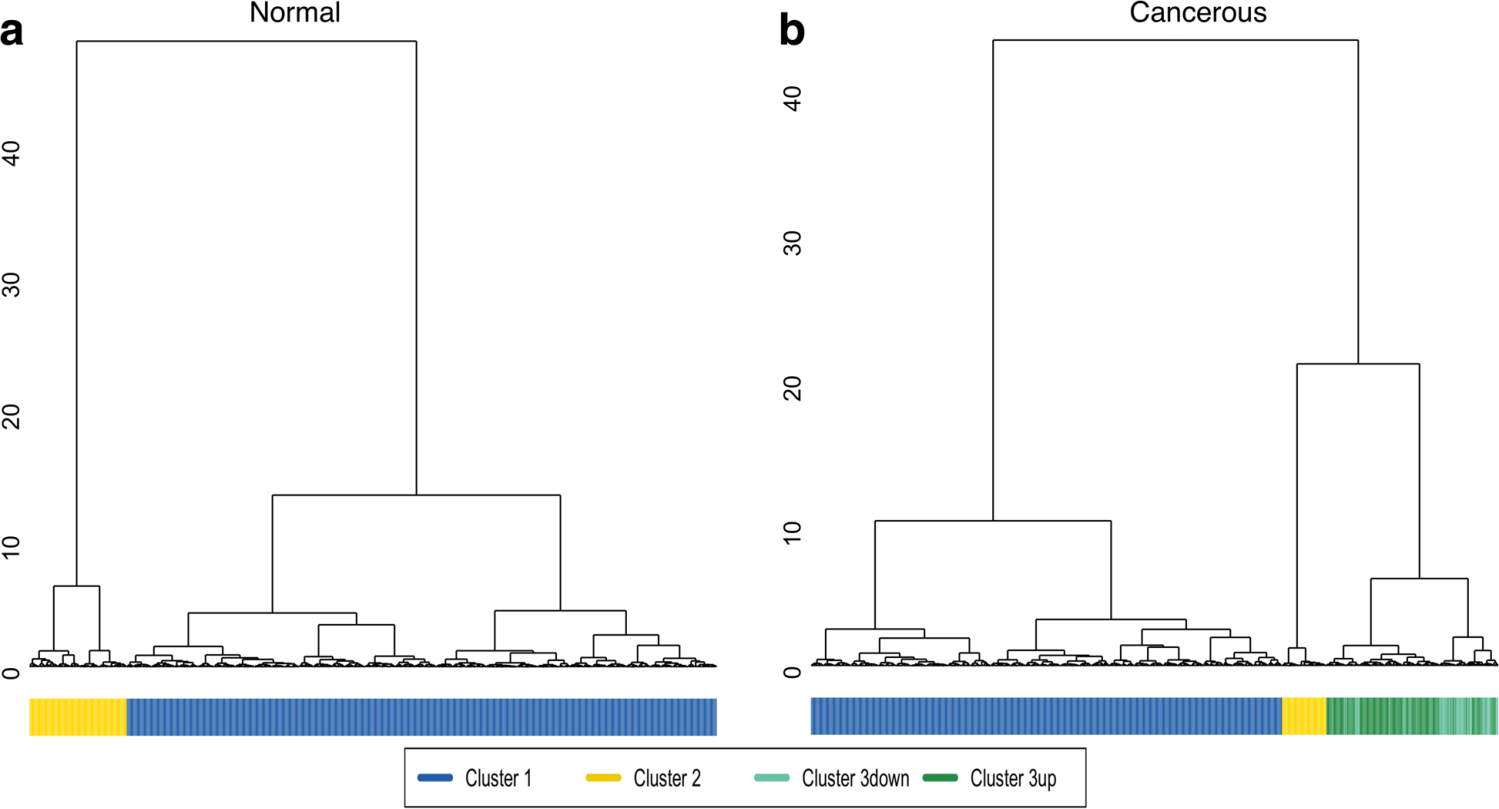
CGI + SS patterns in breast tissues. Hierarchical clustering of CGI + SS DNA methylation patterns for breast normal tissues (panel **a**) and breast cancerous tissues (panel **b**) using DTW as a distance metric and a “Ward” linkage. The colorbar represents the clusters association (blue for hypo-methylated cluster 1, yellow for cluster 2, dark green for cluster 3up, light green for cluster 3down)

**Fig. 2:**
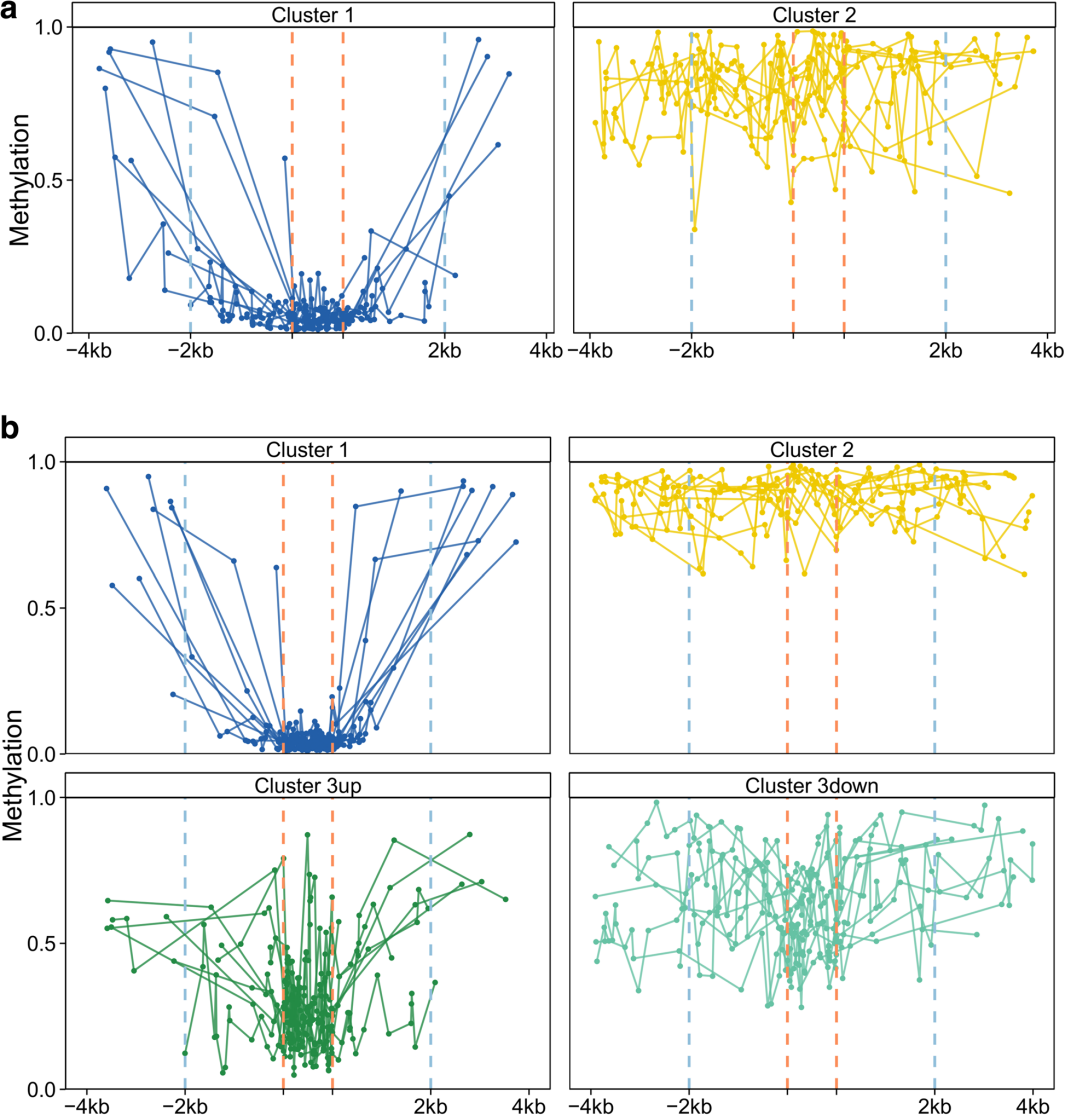
Characteristic profiles for each clusters. Visualization of the CGI + SS DNA methylation signatures as condensed profiles from the 10 medoids profiles for each clusters in breast normal (panel **a**) or cancerous (panel **b**) tissues. The two orange dashed lines represent the normalized 1kb long CGI region while the two blue lines represent the 2kb limit between shores and shelves regions

To assess the diversity of CGI + SS signatures across genes, we perform an unsupervised classification of all signatures for each of the 6 types of samples, using Ward hierarchical clustering. Since different CGI + SS may contain a different number of GpG probes, we use a specific distance based on dynamic time warping to compare signatures of different lengths. Figure 1a shows the CGI + SS clustering obtained for signatures measured on normal samples from breast samples. Similar figures were obtained for lung (Additional file 1a) and colon samples (Additional file 2a). We observe two clusters, which are largely conserved across the 3 tissues of origin (Table 2). To clarify the types of signatures captured by each cluster, we represent on a standardized CGI + SS *x*-axis the 10 medoid CGI + SS signatures for each cluster and each tissue (Fig. 2a, Additional files 3a and 4a). We clearly observe that the large cluster 1, which contains about 90 % of all CGI + SS, corresponds to hypo-methylated islands with hemi-methylated CGI shores and hyper-methylated CGI shelves, while the smaller cluster 2 contains about 10 % of CGI + SS which are fully hyper-methylated. A closer look at cluster 1 shows that, in some cases, the variation of methylation between islands and shores is unclear, in the sense that some shores are fully hypo-methylated. As CGIs, shores and shelves regions are delimited based on somehow arbitrary criteria, a systematic analysis of these signatures could lead to a refinement of currently accepted boundaries.

**Table 2:** Concordance analysis of CGI + SS patterns clusters between normal tissues

Clusters	Colon	
Breast	1	2
1	1560	9
2	113	145
Clusters	Lung	
Colon	1	2
1	1610	7
2	63	147
Clusters	Breast	
Lung	1	2
1	1549	20
2	68	190

**Fig. 3:**
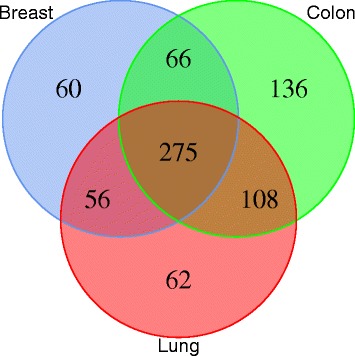
Stability of the cancerous-specific cluster between tissues. Venn diagram representing the intersection of the CGI + SS associated with cancerous-specific cluster 3 for each tissue

Performing the same unsupervised classification independently on signatures obtained from the three types of cancerous tissues leads to different results, with the apparition of a third stable cluster (Fig. 1b for breast, Additional files 1b and 2b for lung and colon, respectively). Comparing the clusters of normal and cancerous tissues shows that, for all types of tissues, the first two clusters found in cancerous tissues are mostly composed of CGI + SS of the corresponding clusters in normal tissues, while the CGI + SS in the third cluster, specifically found in cancerous tissues, tend to come evenly from both clusters in normal tissues (Table 3). A look at representative signatures of each cluster (Fig. 2b for breast, Additional files 3b and 4b for lung and colon, respectively) confirms that clusters 1 and 2 contain respectively hypo- and hyper-methylated profiles, just like the respective clusters in normal tissues, while cluster 3 contains CGI + SS signatures which are hemi-methylated (Additional file 5). Separating the CGI + SS in cluster 3 into sub-clusters “3up” and “3down”, depending on whether they are in cluster 1 or 2 in normal tissues, we further see that the level of methylation of CGI + SS signatures in the “3up” sub-cluster tends to be lower than the level of methylation of CGI + SS signatures in the “3down” sub-cluster (5 to 8 fold decrease). Interestingly, cluster 3 is mostly conserved between tissues (Fig. 3), suggesting that these epigenetic variations might be associated with a tissue-independent carcinogenesis process.

**Table 3:** Concordance analysis of CGI + SS patterns clusters from normal to cancerous tissues. Each table represents the concordance of clusters between normal and cancerous clustering analysis

Breast	Normal	
Cancerous	1	2
1	1231	21
2	9	109
3	329	128
Lung	Normal	
Cancerous	1	2
1	1128	12
2	18	168
3	471	30
Colon	Normal	
Cancerous	1	2
1	1112	11
2	13	106
3	548	37

In summary, this global analysis of methylation signatures suggests the existence of four types of CGI + SS largely conserved across tissues: the majority of them remains hypo-methylated on the CGI and hyper-methylated on the shores and shelves in normal and cancerous tissues (cluster 1); a minority is hyper-methylated in normal and cancerous tissues (cluster 2); finally, a fraction of CGI + SS signatures is hypo-methylated in normal tissues and partly methylated in cancerous tissues (cluster 3up), while another fraction is hyper-methylated in normal tissues and partly methylated in cancerous ones (cluster 3down). To clarify whether these four categories or CGI + SS are associated to particular biological functions, we performed a gene functional enrichment analysis [26] of the genes associated to the CGI + SS in each of the four categories, for each tissue. Results are shown inAdditional file 6. Restricting ourselves to Gene Ontology (GO) biological processes associated to at least 20 genes, we found that the large cluster 1 is mostly enriched in genes involved in metabolic processes, while the cancer-specific cluster 3up is enriched in genes involved in developmental processes. There was no significant functional enrichment for genes in cluster 2 and 3down.

### Cancer-specific methylation does not repress gene expression but instead targets genes lowly expressed in normal tissues

**Fig. 4:**
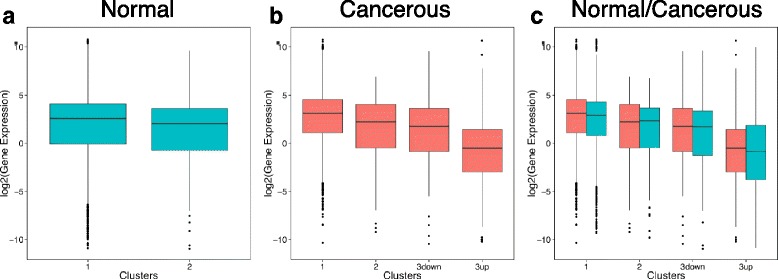
Distribution of gene expression in different clusters for in breast tissues. Gene expression distribution for genes based on the cluster assignment of their associated CGI + SS. Panel **a** Gene expression distribution in normal breast tissues shows a slight repression for genes associated with cluster 2 (hyper-methylated CGI + SS profiles). “Ref” represents the genome-wide gene expression distribution Panel **b** Gene expression profiles in cancerous breast tissues shows high repression for genes associated with cluster 3 and specifically cluster “3up” (hemi-methylated CGI + SS profiles). Panel **c** Gene expression profiles in both normal and cancerous breast tissues using the cluster assignement in cancerous tissues shows that genes associated with cluster “3up” in cancerous tissues define a cluster of genes already repressed in normal tissues

CGI methylation is often associated with gene expression silencing. We therefore assess whether the CGI + SS clusters defined above, corresponding roughly to lowly methylated (clusters 1), highly methylated (cluster 2) or partially methylated in cancer (cluster 3) CGI + SS, are associated with different mean levels of gene expression. In normal breast tissues, we indeed observe that genes near hypo-methylated islands in cluster 1 are slightly but significantly less expressed than genes near an hyper-methylated islands in cluster 2 (Fig. 4a, *P*_*Breast*_=0.02). There is however no significant difference between the two clusters in normal lung tissues (Additional file 7a, *P*_*Lung*_=0.39), and we could not test the hypothesis on normal colon tissues since we have none with both methylation and expression data (Table 1). In cancerous samples, we observe that genes near a CGI + SS in the cancer-specific cluster 3 have a significantly lower expression than other genes (Fig. 4b, Additional files 7b and 8, *P*_*Breast*_,*P*_*Lung*_,*P*_*Colon*_< 10^−16^), particularly for the genes near a CGI + SS in the “3up” cluster. As genes in the “3up” cluster are hypo-methylated in normal tissues, this could suggest that their cancer-specific methylation is a way to repress their expression in cancer. However, a closer look at the expression of these genes in normal tissues (Fig. 4c, Additional file 7c) shows that they are already lowly expressed in normal tissues. This suggests that instead of activating CGI methylation to silence to genes, cancer cells instead activates CGI methylation of hypo-methylated genes which are already lowly expressed in normal tissues.

### Cancer-specific methylation is an independent predictor of patient survival in breast cancer

Our analysis so far compares CGI + SS in terms of their mean methylation across a set of samples and does not take into account between-sample variations. CGI + SS associated with cluster 1 (resp. 3) are hypo- (resp. hyper-) methylated on average, which indicates that there is little to no variations between samples. However, signatures of CGIs in the cancer-specific cluster 3 are partly methylated, which can either hide the fact that they are hemi-methylated for most cancerous samples, or that they are highly variable between samples. We therefore assess whether the partial methylation of CGI + SS signatures in cluster 3 is related to an overall increase (for cluster 3up) or decrease (for cluster 3down) in methylation for all or most of the patients, or if this it is caused by a subset of patients that become hyper- (resp. hypo-)methylated for these CGI + SS.

**Fig. 5:**
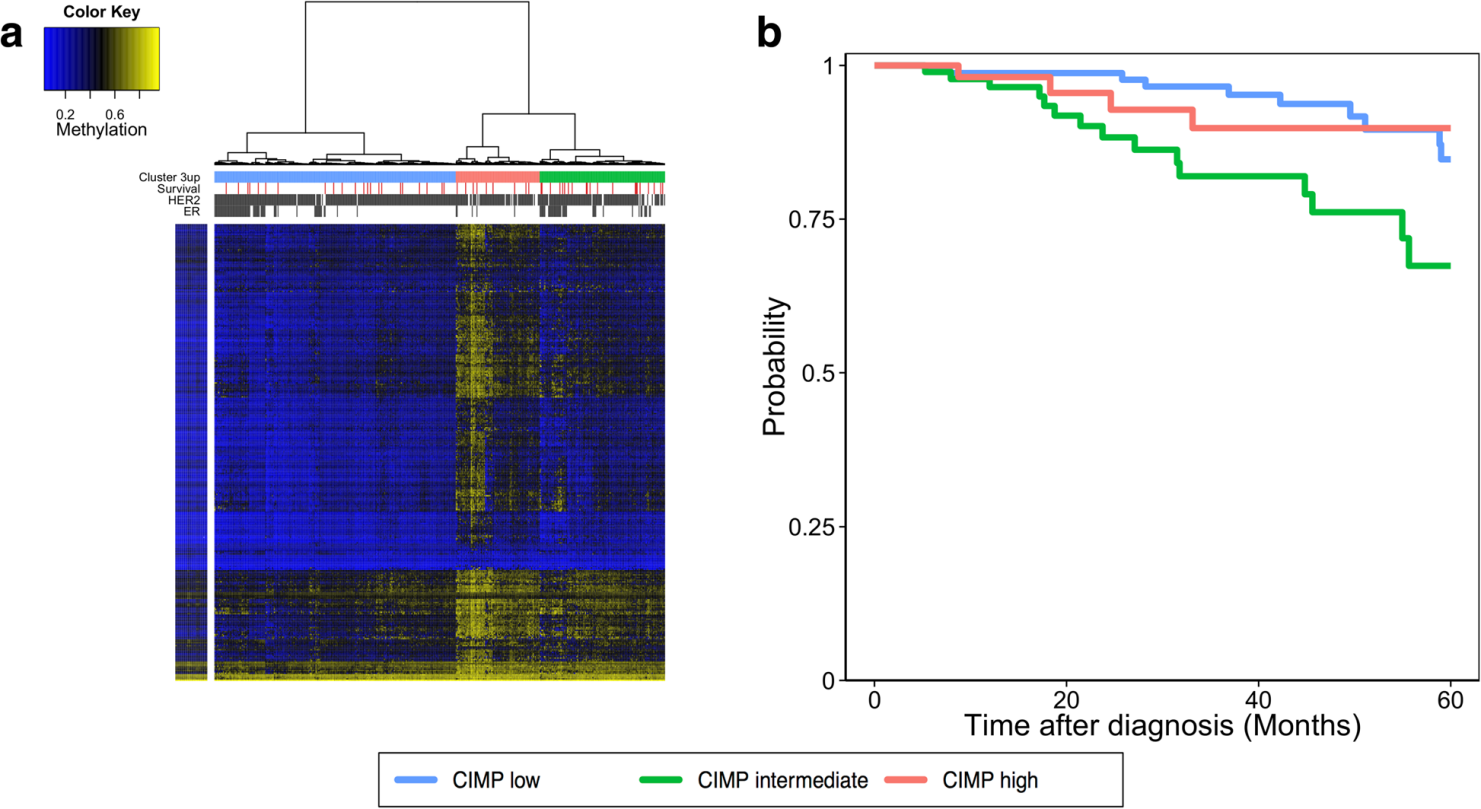
Cluster 3up methylation is a predictive factor for survival of patients in breast cancer patients. Panel **a** Hierarchical clustering of breast cancer patients given the average methylation level of all the CGI + SS associated with cluster 3up. The row color bar represents the average methylation level for the same CGI + SS in healthy breast tissues. The column color bar gives clinical information about the patients such as ER and HER2 statuses (grey for negative and white for positive), survival information (white for positive overall survival within 5 years and red for death within 5 years). The top row of the column color bar represents the three classes distinguished by methylation profiles in cluster 3up (blue for cluster 3up “low”, green for cluster 3up “intermediate” and pink for cluster 3up “high”). Panel **b** Kaplan-Meier estimate of breast cancer patient survival given the cluster 3up class (blue for cluster 3up “low”, green for cluster 3up “intermediate” and pink for cluster 3up “high”) shows that cluster 3up “intermediate” patients have a significantly higher risk of death within 5 years than either cluster 3up “low” or “high” patients (Log-rank, *p* = 0.01)

For that purpose, we first summarize the methylation of each CGI + SS on each breast cancer sample by a single value, the average methylation of the probes in the CGI + SS. We then represent each sample by the vector of methylation values of the CGI + SS in cluster 3up, and perform a Ward hierarchical clustering of the cancerous samples based on this representation. The resulting clustering is shown in Fig. 5a, where in addition we indicate the ER +, HER2 and survival information for each patient. We observe that the distribution methylation values is very bimodal, and that the hyper-methylation of a given CGI + SS from cluster 3up generally happens in a subset of patients only. Interestingly, we see that the same subset of patients tends to be simultaneously hyper-methylated for all CGI + SS from cluster 3up, suggesting that hyper-methylation of these islands is a characteristics of a subset of the tumors. This allows us to divide the set of breast cancer patients in three clusters given the level of methylation in cluster 3up as either “low”, “intermediate”, or “high” (Fig. 5a). Interestingly, distinguishing patients given the level of methylation from the CGI + SS in cluster 3up is significantly predictive of the patient survival (Fig. 5b, log-rank, *p*=0.01). Surprisingly, the cluster with the lowest survival is the “intermediate” cluster encompassing a portion but not all of the triple negative breast cancers (65 % in cluster 3up “low”, 32 % in cluster 3up “intermediate” and only 3 % in cluster 3up “high”). A multivariate Cox proportional hazards regression model fitted with available clinical parameters (tumor size, lymph node status, hormone receptor status, HER2/NEU status and patient’s age) further shows that this stratification of patients based on the methylation level of genes in cluster 3up adds prognostic value independently of other clinical features (Table 4, Additional file 9). These results support the existence of a CpG island methylator phenotype (CIMP) as introduced by Toyota et al. [25] that is clinically relevant to assess the survival of patients. More importantly, they suggest that low survival might not be associated with a positive or negative CIMP, but with an intermediate phenotype termed as CIMP-low [27].

**Table 4:** Multivariate Cox regression analysis including the level of methylation in the cancer-specific cluster “3up” in addition to significant clinical variables for breast cancer

Clinical variable (Reference)	HR (95 % CI)	*p*-value
Cluster 3up (Low vs intermediate)	3.44 (1.44–8.23)	0.007
Cluster 3up (Low vs high)	1.92 (0.50–7.34)	0.34
(ER,HER2) (-/- vs +/-)	0.37 (0.15–0.88)	0.026
(ER,HER2) (-/- vs -/+)	1×10^−8^ (0–Inf)	1
(ER,HER2) (-/- vs +/+)	0.53 (0.09–2.94)	0.46
Lymph Node (Negative)	4.51 (1.63–12.44)	0.004

A similar analysis on CGI + SS associated with cluster 3down is less conclusive, and does not clearly cluster patients in separate clusters (Additional file 10). A lack of sufficient survival data for colon and lung tissues prevented a similar analysis for these tissues.

### Methylation of CpG in the 3’ region outside the CGI is the most correlated with gene expression

Our analysis so far compares CGI + SS to one another, by looking at their average methylation profiles across collections of samples. We found no clear evidence for a correlation between mean methylation level of a CGI and mean expression level of the corresponding genes, but this may be due to the fact that many other factors impact the expression level of a gene, including biological and technical ones. Another way to assess how methylation impacts expression is to look, for each given gene, how variation in expression across samples correlates with variations in methylation of nearby CGIs. For each set of samples (split by tissue of origin and normal/cancerous state), we measure the strength of association between methylation and expression for each gene by computing a predictive goodness of fit *R*^2^ which represents the level of gene expression variation explained by CGI + SS methylation variation (see Materials and methods). This coefficient is calculated either when the CGI + SS methylation status is summarized by the mean methylation values of all the probes, or by using the full CGI + SS methylation information of each probe.

**Fig. 6:**
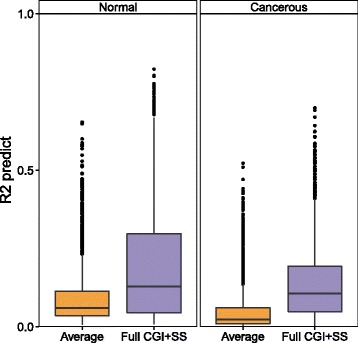
Impact of DNA methylation in gene expression prediction. Predictive power distribution of DNA methylation for gene expression using either the average CGI methylation and least squares (*orange*) or the full CGI + SS profile and lasso regression (*purple*) shows that a more complex model allows to better predict gene expression variations in both normal and cancerous tissues

We observe that the full CGI + SS methylation profile is predictive of gene expression for a subset of genes in each dataset, and that this predictive power is significantly higher than using only the average CGI + SS methylation (Fig. 6, Additional files 11a, b, *P*_*Breast*_< 10^−16^, *P*_*Lung*_=1.3×10^−16^, *P*_*Colon*_=3.2×10^−5^). We provide in Table 5 the list of the top 50 genes based on their predictive score in cancerous breast tissues and similar lists for normal breast, lung and colon tissues in Additional file 12. Among the 2,374 genes studied, 139 genes are associated with more than one CGI + SS. For these genes, the predictive power is computed using the CGI + SS closest to the TSS. Using all the CGI + SS for these genes do not yield significant improvement over taking only the CGI + SS closest to the TSS except for breast tissues (*P*_*Breast*_=0.003, *P*_*Lung*_=0.15, *P*_*Colon*_=0.62). We also observe no association between the predictive goodness of fit *R*^2^ and the CGI + SS clusters described above (*P*_*Breast*_=0.48, *P*_*Lung*_=0.47, *P*_*Colon*_=0.44).

**Table 5:** Genes regulated by methylation in breast cancer

Gene	Score
*DQX1*	0.6993144
*IRS2*	0.6920052
*GPSM3*	0.6698851
*FOXC1* ^*†*^	0.6428115
*PSMB9*	0.6242704
*HOXC10* ^*†*^	0.6233063
*NDRG2*	0.6230449
*MAPT*	0.6078226
*STC2*	0.6064161
*ZNF502* ^*†*^	0.5859661
*PTPRCAP*	0.5834326
*SCAND3*	0.5832075
*SLC1A4*	0.5801594
*TAP1*	0.5763637
*DBNDD2*	0.5650488
*OTX1* ^*†*^	0.5648463
*TCF7* ^*†*^	0.5618545
*LY6G6C*	0.5618097
*FERMT3*	0.5602340
*ZIC4* ^*†*^	0.5595657
*HLA-B*	0.5565054
*GDF9*	0.5517472
*SOX9* ^*†*^	0.5513052
*CELSR1*	0.5502329
*SYS1-DBNDD2*	0.5490248
*HLA-E*	0.5490118
*CYP1B1*	0.5411610
*RUNX3* ^*†*^	0.5406337
*KIAA1949*	0.5379938
*RIPK4*	0.5313998
*TPPP2*	0.5305543
*HLA-F*	0.5304392
*PPP1R3C*	0.5293955
*HOXB5* ^*†*^	0.5287862
*CELSR3*	0.5272638
*B3GNT5*	0.5259399
*ME3*	0.5244800
*TMC8*	0.5231671
*AIF1*	0.5223122
*SLC39A6*	0.5217374
*HOXC11* ^*†*^	0.5122936
*ERBB2*	0.5055868
*TBC1D10C*	0.5038227
*SIM2*	0.5030521
*CAMK2N1*	0.5022371
*RGMA*	0.4996740
*LOC100132215*	0.4979089
*PAX6* ^*†*^	0.4976355
*VANGL2*	0.4960224
*DDHD2*	0.4879724

*Gene*: Top scoring genes ranked by the predictive power of methylation to predict gene expression variation. *Score*: *R* ^2^ score associated. Transcription factors are highlighted with a *†*

**Fig. 7:**
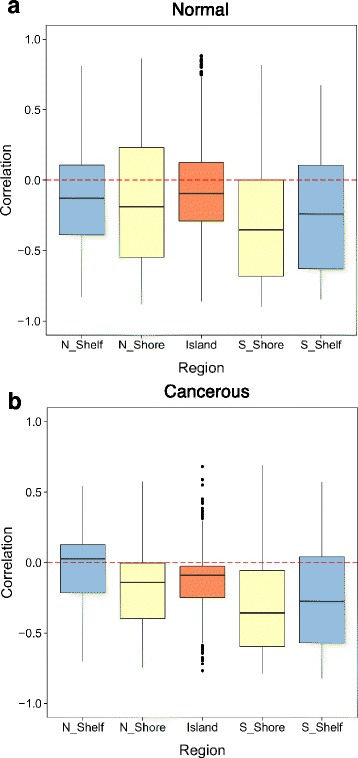
Correlation between individual CpG methylation and gene expression for breast cancer. Distribution of the correlation between individual CpG methylation and gene expression for normal (panel **a**) and cancerous (panel **b**) breast samples. The analysis is restricted to the 50 genes with the largest *R* ^2^ predictive value. Correlations are aggregated by regions in the CGI + SS. We see the strongest association for probes located outside of the CGI, particularly in shores regions, in both normal and cancerous tissues

Since the predictive power of multivariate models based on all CpG probes in a CGI + SS is larger than the predictive power of the mean methylation value only, we now investigate which CpG in a CGI + SS are particularly important predictors of expression. For that purpose, we measure the correlation between the methylation of individual CpG and gene expression for the 50 genes with the largest predictive *R*^2^, and summarize the correlation values based on the position of the probe in the CGI + SS in Fig. 7 for breast samples (Additional file 13 for colon and lung). As expected, we observe overall a negative correlation between methylation and gene expression, and notice that this association is stronger in CGI shores and shelves located in the 3’ region than in the CGI itself. This is coherent with results in [11] stating that variations in the CGI are less critical than variations in proximity regions of the CGI. Performing the same analysis by varying the number of genes selected to compute correlations from 20 to 100 gave similar results.

### Correlation between gene expression and promoter methylation is tissue-specific, and is modified in cancer tissues but overall targets transcription factors

**Fig. 8:**
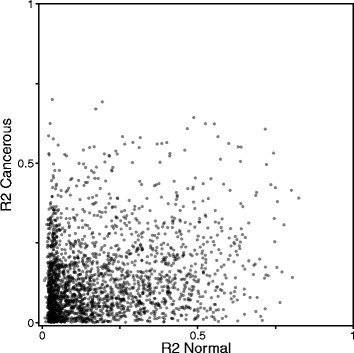
Shift of epigenetic regulation in cancer. Scatterplot between the predictive power of DNA methylation for gene expression in normal and cancerous breast tissues shows little correlation (*R* ^2^=0.04)

Results in the previous section suggest that for a subset of genes, a significant correlation between promoter methylation and gene expression is observed, which may be due for example to a direct regulation of gene expression by promoter methylation. To assess whether this correlation is conserved across tissues, we compare the predictive powers of methylation for each gene when it is computed on normal or cancerous samples from different tissues. As shown on Additional file 14, however, we observe little correlation between the predictive power across tissues in normal and in cancer samples, suggesting that the association between promoter methylation and gene expression is tissue-specific ($R^{2,Normal}_{Breast/Lung}=0.04$, $R^{2,Cancerous}_{Breast/Lung}=0.17$, $R^{2,Cancerous}_{Lung/Colon}=0.07$, $R^{2,Cancerous}_{Colon/Breast}=0.06$). We also observe very little correlation between predictive powers in normal and cancerous tissues, which could suggests a shift of the epigenetic regulation mechanism during cancer development (Fig. 8, Additional file 15, $R^{2}_{\textit {Breast}}=0.05$, $R^{2}_{\textit {Lung}}=6 \times 10^{-7}$).

Many mechanisms besides DNA methylation are involved in gene expression regulation. In particular, transcription factors (TF) play a critical role in the recruitment of RNA polymerase that allows gene transcription [28]. We noticed that the list of the 50 genes with the largest predictive *R*^2^ score in each tissue is significantly enriched in TFs as collected from [29], suggesting that methylation may play an important role in the gene regulatory process of transcription factors (*P*_*Breast*_=0.03, *P*_*Lung*_=3×10^−4^, *P*_*Colon*_=0.02). Using the TF list obtained from [30] yields similar conclusions, as well as varying the number of genes selected from 20 to 100.

### Copy number variations in cancer is an independent factor correlated with gene expression

**Fig. 9:**
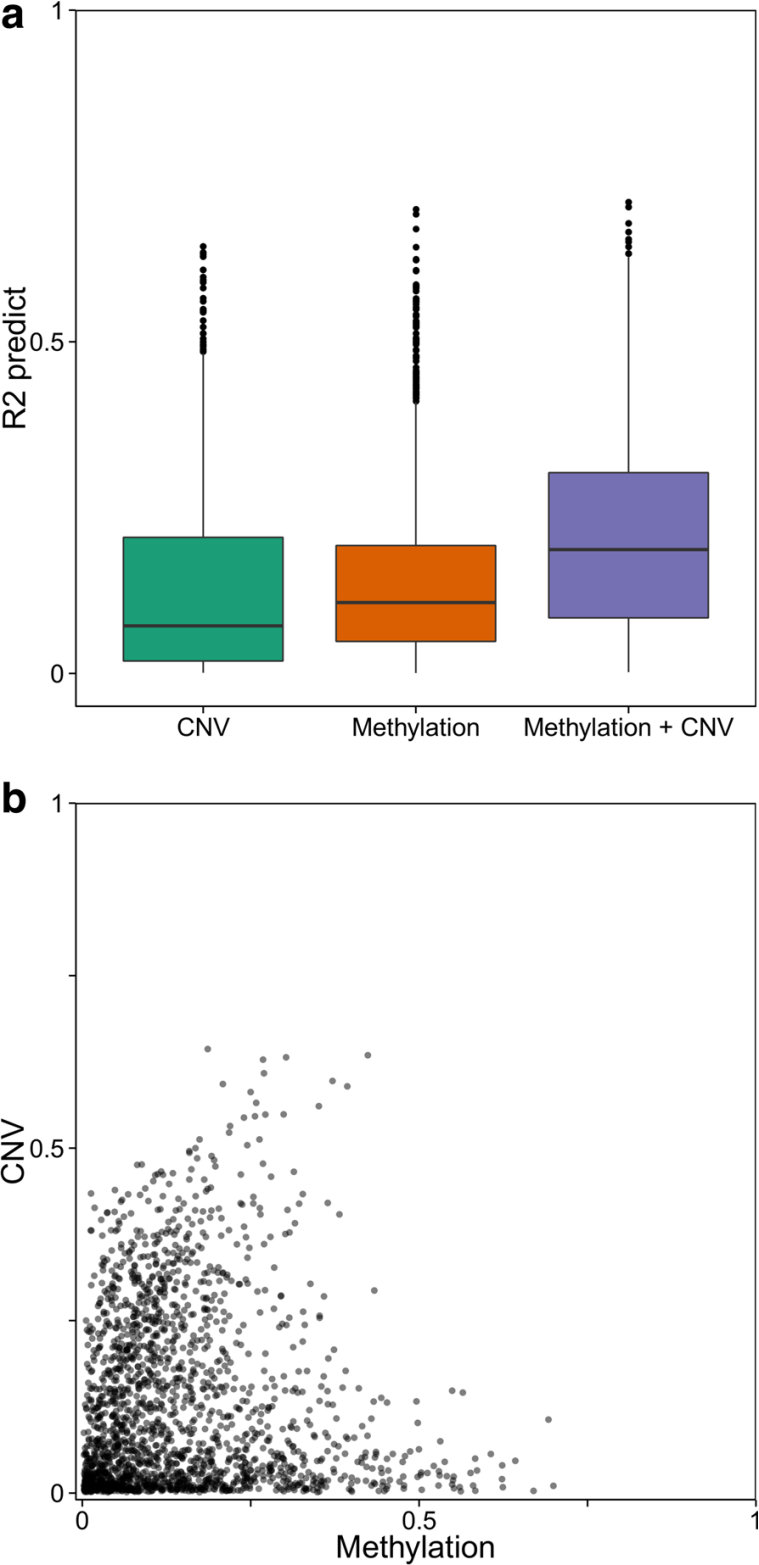
Association between predictive power of methylation and CNVs. Panel **a** Predictive power distribution using either CNV data only with least squares, DNA methylation data only with lasso regression or both CNV and DNA methylation data with lasso regression. Combined methylation and CNV information yield significantly higher predictive power (*P*< 10^−16^). Panel **b** Scatterplot of predictive power using DNA methylation only and copy number information only shows that both regulation mechanisms operate exclusively on genes (*R* ^2^=6×10^−4^)

In cancer, aberrant DNA copy number variations (CNVs) can have an important impact on gene expression phenotypes [31]. Since genome-wide DNA copy number information is available for all samples analyzed in this study, we now perform an integrated analysis combining methylation, DNA copy number and gene expression. We compute a predictive goodness of fit *R*^2^ to represents the power of DNA copy number information alone to predict gene expression, on the one hand, and a multidimensional regression model combining both the full CGI + SS DNA methylation information and the DNA copy number information, on the other hand. We observe that combining methylation and copy number information leads to significantly better results in predicting gene expression than taking each information separately (Fig. 9a, Additional files 16–17a, *P*_*Breast*_< 10^−16^, *P*_*Lung*_< 10^−9^,*P*_*Colon*_< 10^−8^). Moreover, correlation analysis between predictive scores using DNA methylation only, on the one hand, and predictive scores using CNVs only, on the other hand, shows very little correlation ($R^{2}_{\textit {Breast}}=7 \times 10^{-4}$, $R^{2}_{\textit {Lung}}=1 \times 10^{-4}$, $R^{2}_{\textit {Colon}}=1 \times 10^{-3}$, Fig. 9b, Additional files 16–17b). This suggests that both methylation and DNA CNVs are important and non-redundant predictors of gene expression variation.

## Discussion

DNA methylation is a well-described process in normal development and is critical in specific gene expression regulations such as X-chromosome inactivation, genomic imprinting and tissue developpment [2–5]. Since aberrant hyper- and hypo-methylation have also been frequently observed in cancer, it has been often argued that activation of oncogenes or repression of tumor suppressor genes could be caused by these epigenetic variations [6].

In the present study, we assessed the existence of characteristic CGI + SS DNA methylation signatures in normal tissues and showed a weak association between the hyper-methylated signature and gene expression repression. A similar study in cancerous tissues showed the existence of a cancer-specific signature highly associated with repressed genes. However, the corresponding genes are already highly repressed in normal tissues, questioning the causal impact of methylation in gene expression regulation, as already observed [18, 19, 21].

Using regression methods we analyzed whether differences between CGI + SS methylation across samples - independently of signatures - are predictive of gene expression variation. We showed that for certain genes, expression variations across samples can be well predicted from DNA methylation and that these genes are not associated with cancer-specific methylation patterns. We also showed that using the full CGI + SS methylation profiles in a multidimensional regression framework yields better predictive power than summarizing the methylation of a CpG island by one mean value, as done in previous studies [32]. Looking at probewise methylation correlation with gene expression for the top scoring genes, we observed that the impact of a CpG methylation on gene expression is largely dependent on its location in or near the island, and that CpGs located outside of CGIs have a bigger impact on gene expression variations than CpG located within the CGI, in accordance with [11, 33]. The impact of CGIs located outside of promoter regions, such as intragenic CGIs is still unclear as it does not seem to contribute significantly to global gene expression regulation. Yet, a few studies point at their potential role in modulating alternative promoters [34] or in long-range regulation [35].

Reproducing this methodology on different datasets allowed us to compare the variations of gene expression regulation by methylation in normal and cancerous tissues but also between different types of tissues. Our results suggest that genes targeted by methylation are not only very different between different normal tissues, but more importantly that they are very different between normal and cancerous samples of a given tissue suggesting a shift of epigenetic regulation between normal and cancerous tissues. Recently, hydroxymethylation of cytosines (hmC) has been shown to be significantly present in mammalians cells [36] and methylation data generated with Illumina arrays, as done here, are not able to distinguish methylation (mC) from hmC [37]. However, hydroxymethylation is significantly less observed in cancer tissues [38, 39]. It is therefore likely that the epigenetic information measured here is indeed cytosine methylation.

In addition, the association between DNA methylation and other important regulation mechanisms widens our understanding of the role of methylation in the whole gene expression regulation process. While TFs are essential for controlling gene expression, we showed that their activation itself is significantly associated with DNA methylation markers, highlighting the critical role of methylation in the regulatory process. CNVs have been widely analyzed as a source of genetic variation that plays an important role in complex phenotypes such as cancer [31, 40]. While CNV contribution has been characterized on a genome-wide scale, the link with other regulation mechanisms, particularly DNA methylation, is still unclear [41, 42]. We showed that the impact of both processes in gene expression regulation seems to be non-redundant. The relatively large dataset size gives us confidence in the statistical validity of the results, which are however limited to a fraction of the total genes because of uneven coverage. Methylome sequencing has already been performed and also supports the complexity of methylation patterns but is still limited to very small datasets [32]. Undoubtedly, larger methylome datasets available in the near future will further improve our understanding of the role of DNA methylation in gene expression regulation.

## Conclusions

In summary, this study suggests that promoter methylation profiles can be summarized with a few characteristic profiles that we refer to as CGI+SS methylation signatures. In cancer, we observe an epigenetic reprogramming that leads to the apparition of a cancer-specific CGI+SS methylation signa- ture. However, this epigenetic reprogramming is not associated changes in gene expression, suggesting that this mechanism does not contribute to cancer development via direct inhibition of gene expression through promoter hypermethylation. On the other hand, we observe that genes which demonstrate high correlation between methylation variations and gene expression variations differ from normal to cancer- ous tissues. This suggests that in cancer, the association between gene expression and promoter DNA methylation is modified.

## Materials and methods

### Patients selection

All data were retrieved from the TCGA data portal. We selected samples from breast, colon and lung adenocarcinomas because large matched datasets were available for methylation, gene expression and copy number profiles. The datasets are detailed in Table 1 and the different institutions that released the data are mentioned in the acknowledgement section.

### Methylation profiling

Methylation profiles were retrieved from level 2 TCGA data. They were obtained with the Illumina HumanMethylation450K DNA Analysis BeadChip assay, which is based on genotyping of bisulfite-converted genomic DNA at individual CpG-sites to provide a quantitative measure of DNA methylation [43]. Following hybridization, the methylation value for a specific probe was calculated as the ratio *M*/(*M*+*U*) where *M* is the methylated signal intensity and *U* is the unmethylated signal intensity. 485,577 CpG methylation levels, associated with 27,176 CGIs and 21,231 genes, were measured as such accross the genome.

Following [11], we considered not only the CGI methylation profile but also included in the analysis proximal regions in the near vicinity (up to 4kb), namely the CGI Shores and Shelves regions in a general CGI + SS methylation profile. As we were interested in the coordinated variations of methylation, we restricted the analysis to CGI + SS profiles containing at least 20 probes which reduced the analysis to 1827 CGI+SS associated with 2,374 genes from the original dataset.

### Gene expression profiling

Gene expression profiles were retrieved from level 3 TCGA data. They were obtained from the Illumina HiSeq RNASeq technology and processed following [44].

### CNV processing

CNVs were retrieved from the level 3 TCGA data infered from Affymetrix SNP6.0 data files in GenePattern following [45]. For each gene, we then obtained the log ratio copy number score as the segmented log ratio score for the interval containing its transcription start site.

### Combined CpG island, shores and shelves pattern analysis

CGI + SS patterns were compared using dynamic time warping (DTW) [46] as it is less sensitive to small variations than the Fréchet distance [47]. Dynamic time warping was originally applied as a speech signal similarity measure and has been applied with success in several other fields including computer vision [48], protein structure matching [49] and time series analysis [50].

A CGI + SS profile *i* can be represented as a couple of vector $(X^{i},Y^{i})=(({x^{i}_{1}},{y^{i}_{1}}),\ldots, ({x^{i}_{n}},{y^{i}_{n}}))$ where ${x^{i}_{k}}$ represents the position of the *k*^*th*^ CpG associated with the CGI + SS and ${y^{i}_{k}} \in \,[0;1]$ represents the mean methylation level for this probe accross a given dataset. For two CGI + SS profiles with respectively *m* and *n* probes, we compute the distance between the two profiles as:

$$ DTW({CGI}_{1}, {CGI}_{2}) = \min_{w \in Path} \sum_{k = 1}^{length(w)} \vert y^{1}_{{w_{1}^{k}}} - y^{2}_{{w_{2}^{k}}} \vert^{2} \,, $$

where a path *w* of length *K* is a pair of vectors $({w_{1}^{k}},{w_{2}^{k}})_{k\in [1:K]}$ in [1;*m*]×[1;*n*] that verifies:

- $({w_{1}^{1}},{w_{2}^{1}}) \in \{1\} \times [1;n] \cup [1;m] \times \{1\}$ (partial initialization)
- ∀*i*∈{1;2}, $w_{i}^{k+1}={w_{i}^{k}}$ or $w_{i}^{k+1}={w_{i}^{k}}+1$ (monotonicity and continuity)
- $({w_{1}^{K}},{w_{2}^{K}}) \in \{n\} \times [1;n] \cup [1;m] \times \{n\}$ (partial boundary condition)

The algorithm is applied for each pair of CGI + SS patterns to obtain a dissimilarity matrix. Ward hierarchical clustering is then performed on this dissimilarity matrix to assess the existence of characteristic patterns amongst the different datasets.

The number of significant clusters is assessed through bootstrapping (*n*_*repeats*_=100) on a random subset of CGI + SS of the initial dataset (*ratio*=80 % of the total number of CGI + SS) following Ben-Hur et al [51]. R code for analysis is available upon request.

### Survival analysis

Overall survival was estimated using the Kaplan-Meier method [52] to compare the survival between the group of patients with a lower level of methylation in the hemi-methylated CGI + SS compared to the group of patients with a higher level of methylation. A multivariate Cox proportional hazards regression model [53] was also fitted to estimate the additional value of this classification as a predictive factor for survival compared to other clinical parameters such as age, tumor size, lymph node status, receptor status and HER2/NEU status.

### Computing gene expression susceptibility to DNA methylation changes

We apply ridge [54] and LASSO [55] multivariate regression methods to predict gene expression using the full CGI + SS methylation profiles as well as univariate least square regression when using only the averaged methylation from the whole CGI + SS profile. Following Acharjee et al. [56], we assess the predictive power of the methylation using the predictive goodness of fit *R*^2^ which represents the squared Pearson correlation between observed and fitted values on an independent dataset. The estimation of the predictive power for each gene is obtained through 3-fold cross-validation averaged over 100 repeats. Parameters for both lasso and ridge regression methods were obtained by minimizing the mean squared error function using nested 3-fold cross-validation on the training dataset. The use of the predictive goodness of fit instead of the classic mean squared error as a score allows to compute a comparable score between different predictions. In particular, the mean squared error is highly affected by the absolute level of gene expression while the *R*^2^ is invariant to scaling. It is also important to note that in this case the *R*^2^ computed for least square regression is a prediction *R*^2^ and not just a goodness-of-fit of the given dataset and therefore provides confidence on the generalization of the score on independent datasets. R code for analysis is available upon request.

## Additional files

Additional file 1
**Hierarchical clustering of average CGI + SS patterns for lung tissues.** (PDF 136 kb)

Additional file 2
**Hierarchical clustering of average CGI + SS patterns for colon tissues.** (PDF 135 kb)

Additional file 3
**Characteristic profiles of CGI + SS clusters in lung tissues.** (PDF 1249 kb)

Additional file 4
**Characteristic profiles of CGI + SS clusters in colon tissues.** (PDF 1311 kb)

Additional file 5
**Example of CGIs with different methylation patterns between normal and tumor samples (full dark blue line=cluster 1 in normal samples, dashed green line=cluster 3up in tumor samples).** The black vertical bars represent the position of the CpG island. (PDF 922 kb)

Additional file 6
**Gene ontology analysis for the genes associated with cancer-specific cluster 3.** (PDF 72 kb)

Additional file 7
**Gene expression patterns for each CGI + SS clusters in lung tissues.** (PDF 286 kb)

Additional file 8
**Gene expression patterns for each CGI + SS clusters in colon tissues.** (PDF 5.18 kb)

Additional file 9
**Clinical impact of cluster “3up”.** Multivariate Cox regression analysis including the level of methylation in the cancer-specific cluster “3up” in addition to all clinical variables available for breast cancer. (CSV 4091 kb)

Additional file 10
**Hierarchical clustering of breast cancer patients based on the average methylation level of CGI + SS associated with cluster 3down.** (PDF 379 kb)

Additional file 11
**Impact of DNA methylation on gene expression prediction.** (PDF 156 kb)

Additional file 12
**Top genes regulated by methylation for breast normal tissues, lung normal and cancerous tissues and colon cancerous tissues.** (CSV 3.48 kb)

Additional file 13
**Methylation association with gene expression by regions.** **Panel A.** Colon cancerous samples. **Panel B.** Lung normal samples. **Panel C.** Lung cancerous samples. (PDF 252 kb)

Additional file 14
**Tissue-specificity of methylation for gene expression regulation in normal and cancerous tissues.** **Panel A.** normal breast and lung samples. **Panel B.** cancerous breast and colon samples. **Panel C.** cancerous colon and lung samples. **Panel D.** cancerous lung and breast samples. (PDF 714 kb)

Additional file 15
**Shift of epigenetic regulation between normal and cancerous lung tissues.** (PDF 135 kb)

Additional file 16
**Specific increase of predictive power for aberrant CNV by including copy number profiles in lung tissues.** (PDF 415 kb)

Additional file 17
**Specific increase of predictive power for aberrant CNV by including copy number profiles in colon tissues.** (PDF 367 kb)

## Abbreviations

CGI: CpG Island
CGI + SS: Combined CpG Island, CGI shores and shelves
CNV: Copy number variation
GRM: Gene regulated by methylation
TF: Transcription factor
DTW: Dynamic time warping
