# Supplementary figures and images for: Changes in correlation between promoter methylation and gene expression in cancer
Source: BMC Genomics. 2015 Oct 28;16:873. doi: 10.1186/s12864-015-1994-2 (PMC4625954; doi:10.1186/s12864-015-1994-2)

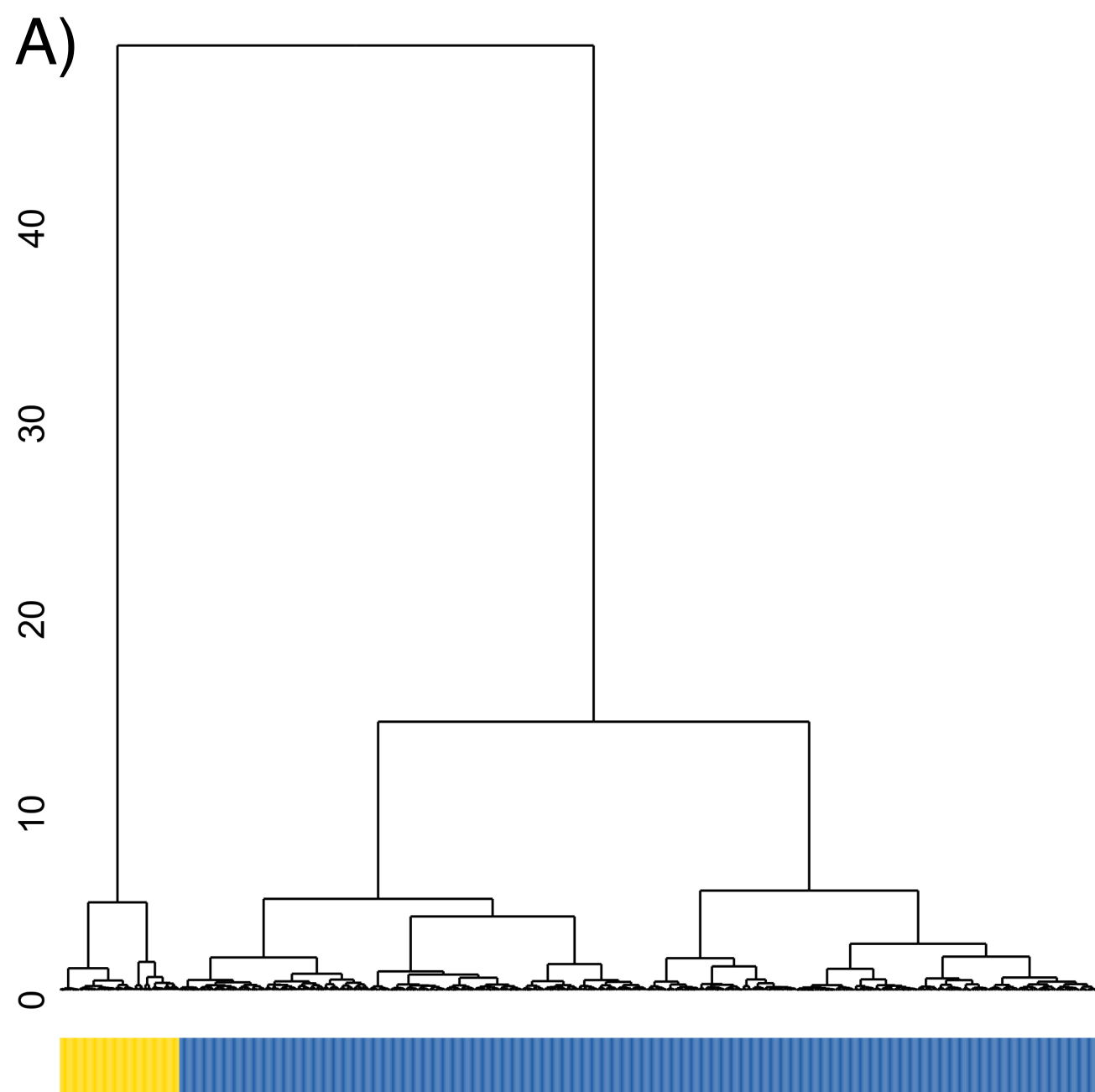

Supplement: Additional file 1 — Hierarchical clustering of average CGI + SS patterns for lung tissues. (PDF 136 kb) [file 12864_2015_1994_MOESM1_ESM.pdf]

A)

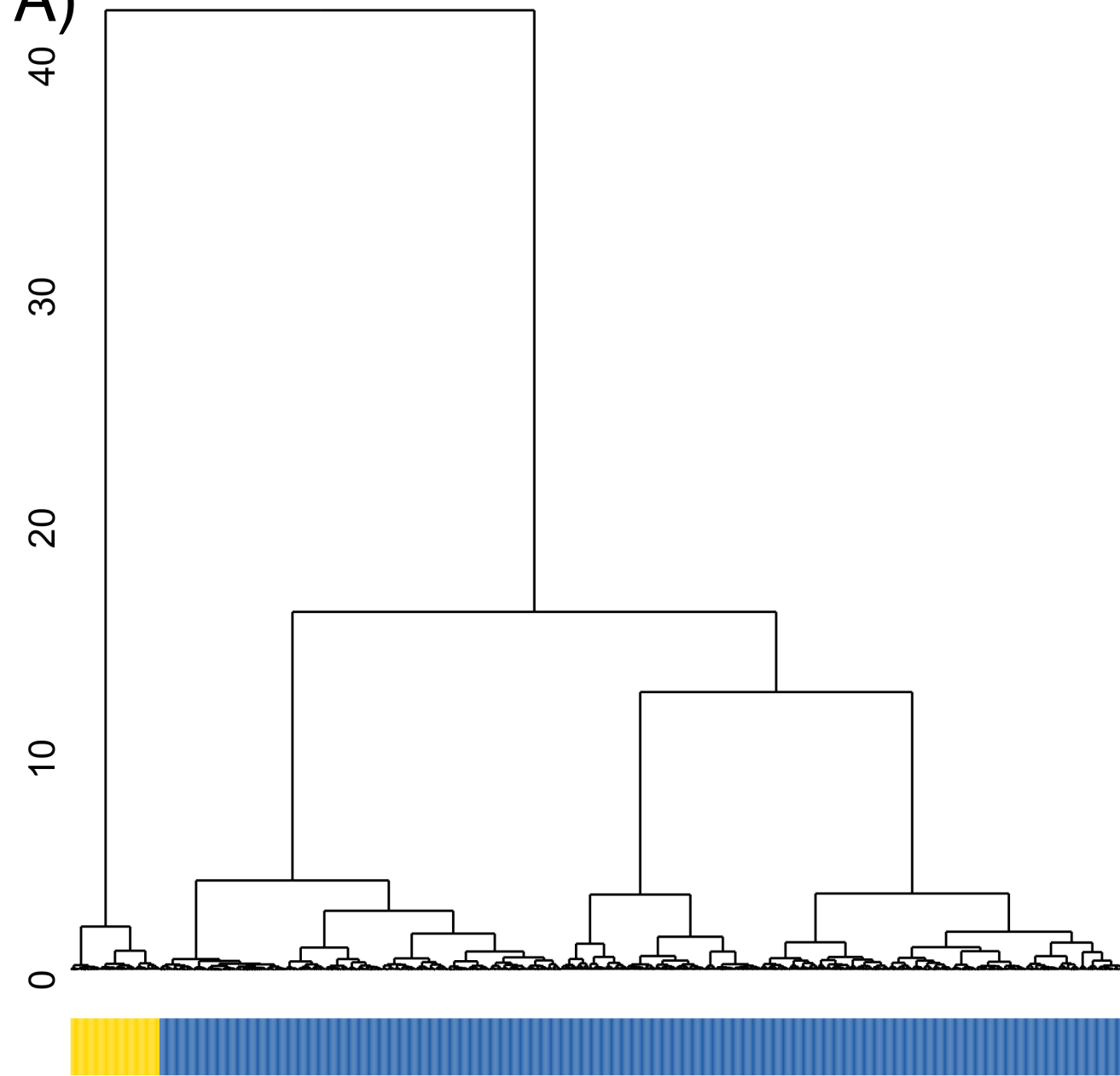

B)

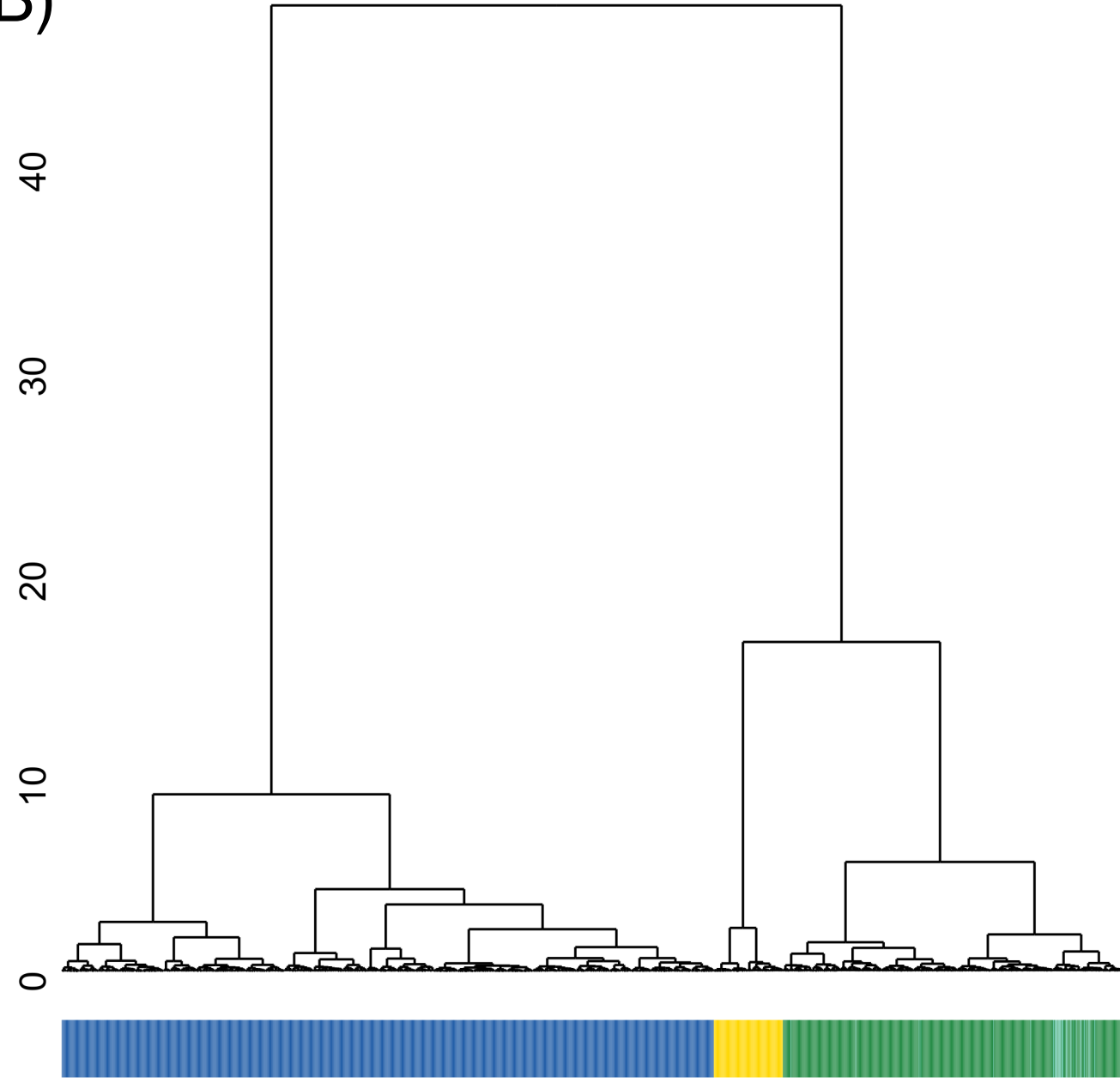

Supplement: Additional file 2 — Hierarchical clustering of average CGI + SS patterns for colon tissues. (PDF 135 kb) [file 12864_2015_1994_MOESM2_ESM.pdf]

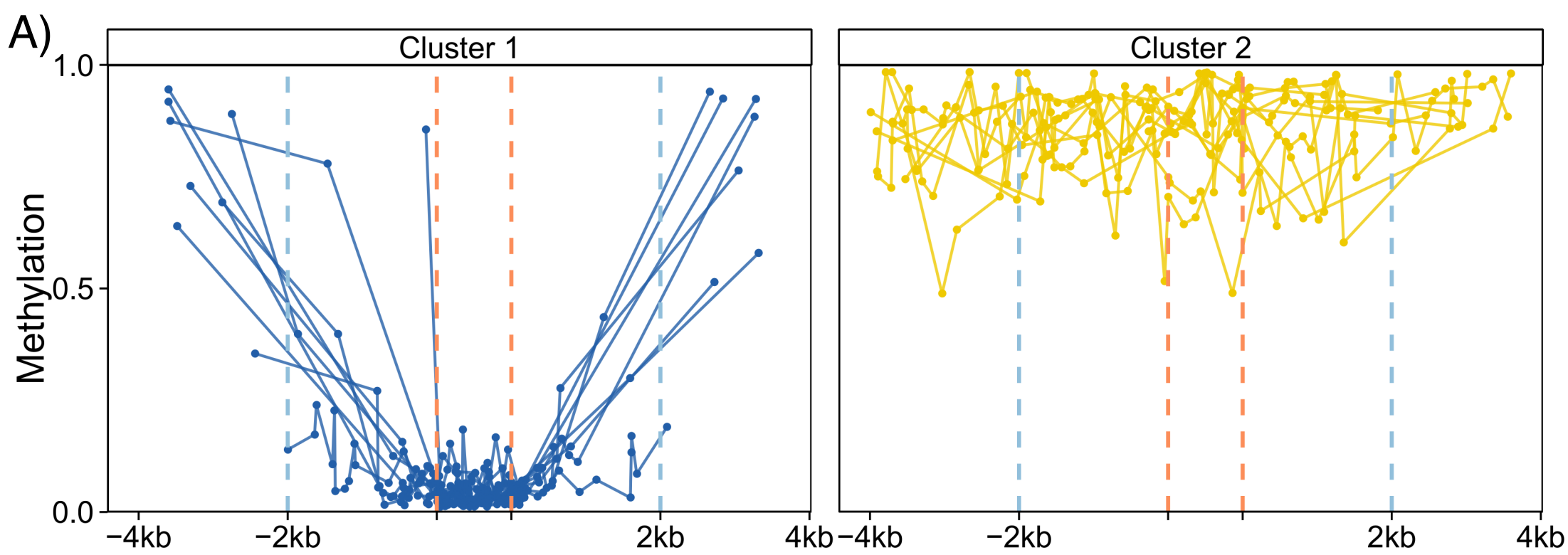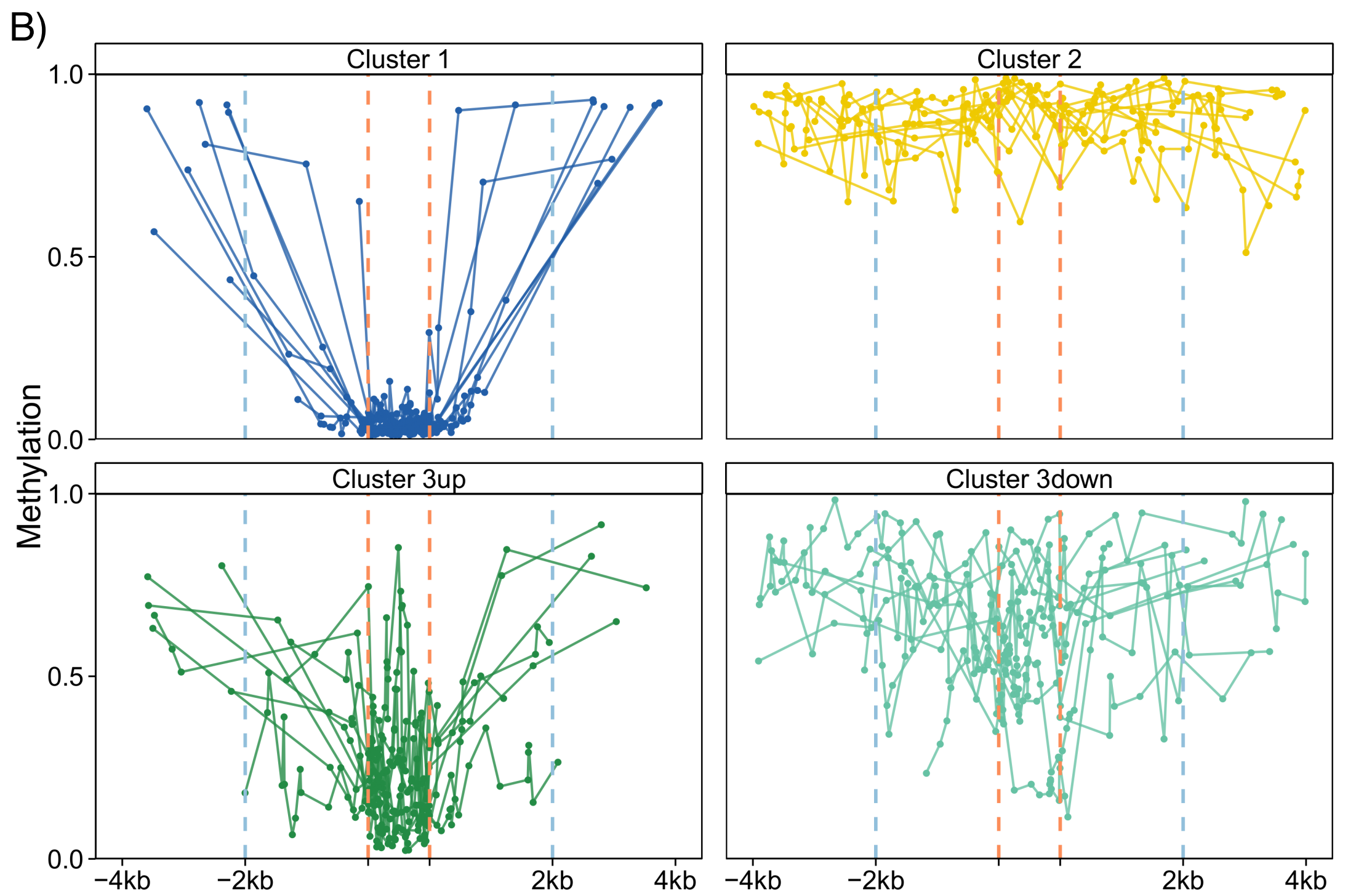

Supplement: Additional file 3 — Characteristic profiles of CGI + SS clusters in lung tissues. (PDF 1249 kb) [file 12864_2015_1994_MOESM3_ESM.pdf]

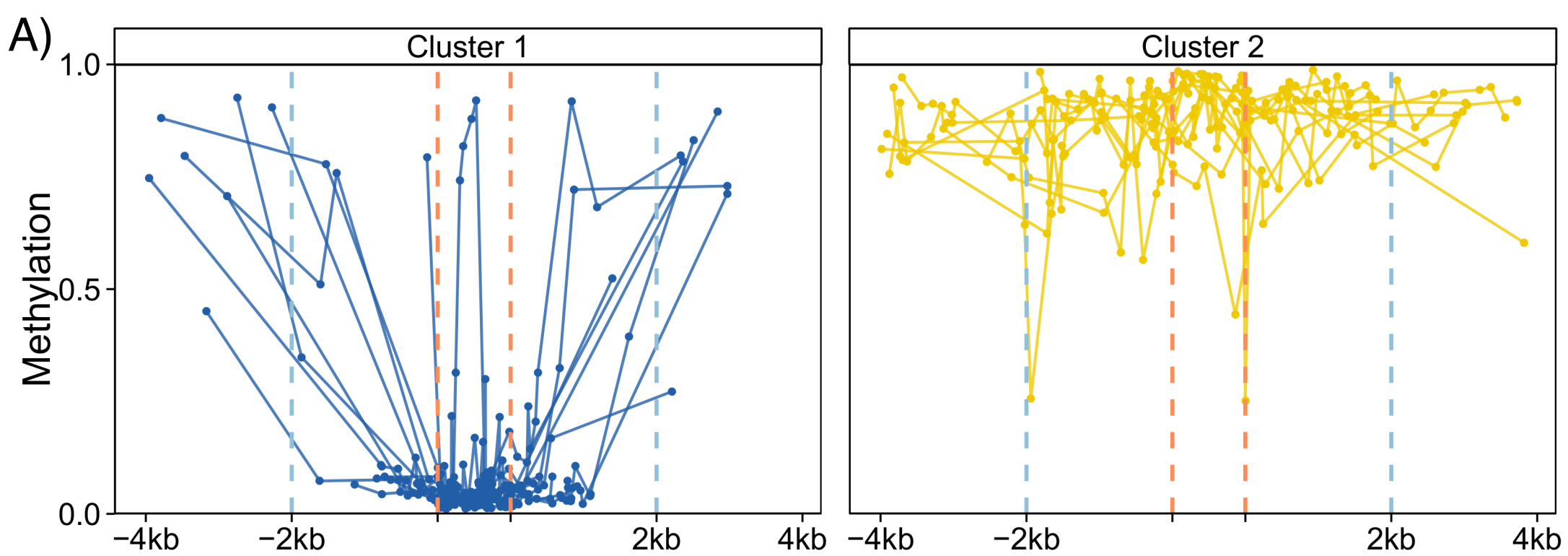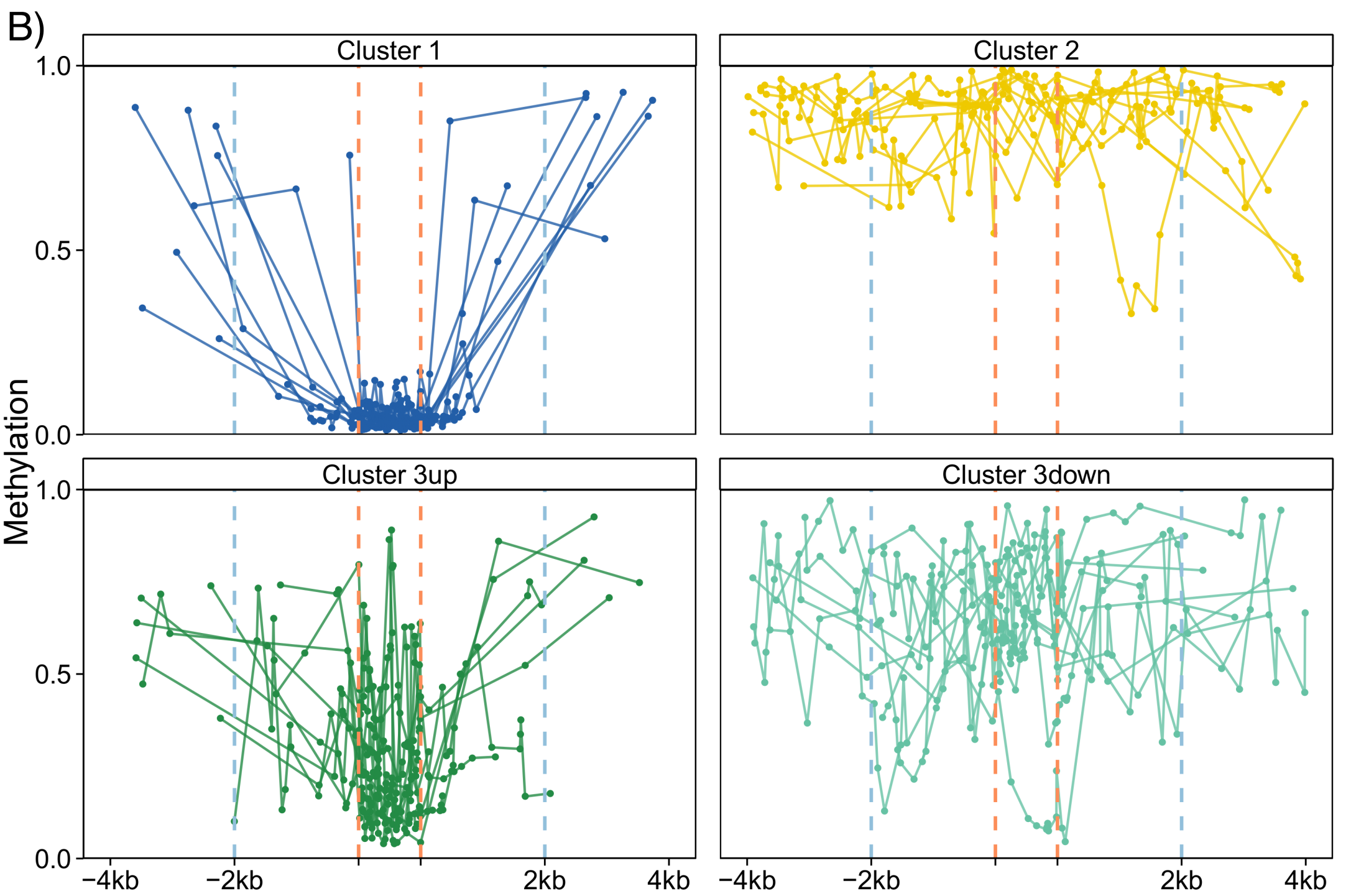

Supplement: Additional file 4 — Characteristic profiles of CGI + SS clusters in colon tissues. (PDF 1311 kb) [file 12864_2015_1994_MOESM4_ESM.pdf]

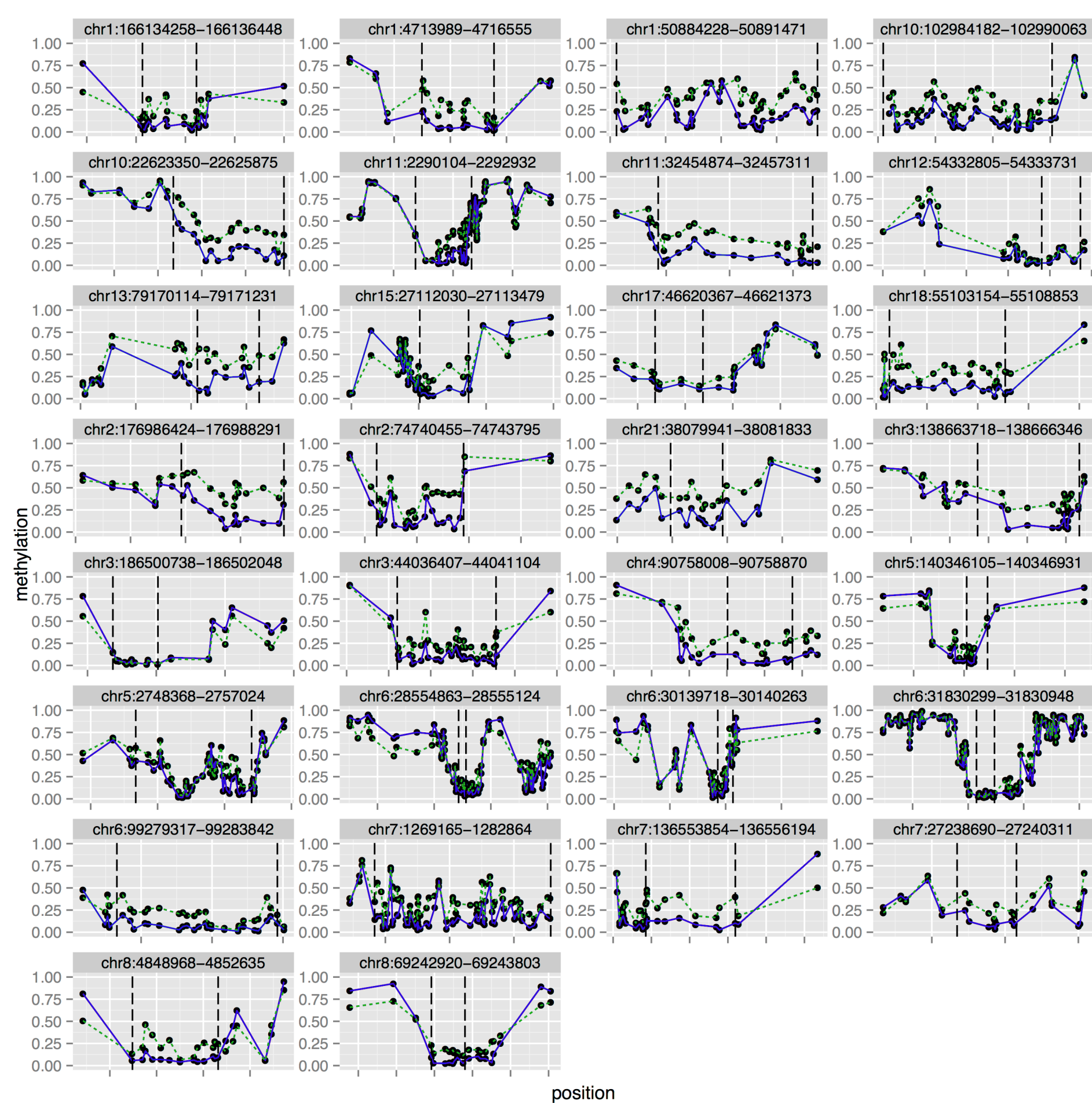

Supplement: Additional file 5 — Example of CGIs with different methylation patterns between normal and tumor samples (full dark blue line=cluster 1 in normal samples, dashed green line=cluster 3up in tumor samples). The black vertical bars represent the position of the CpG island. (PDF 922 kb) [file 12864_2015_1994_MOESM5_ESM.pdf]

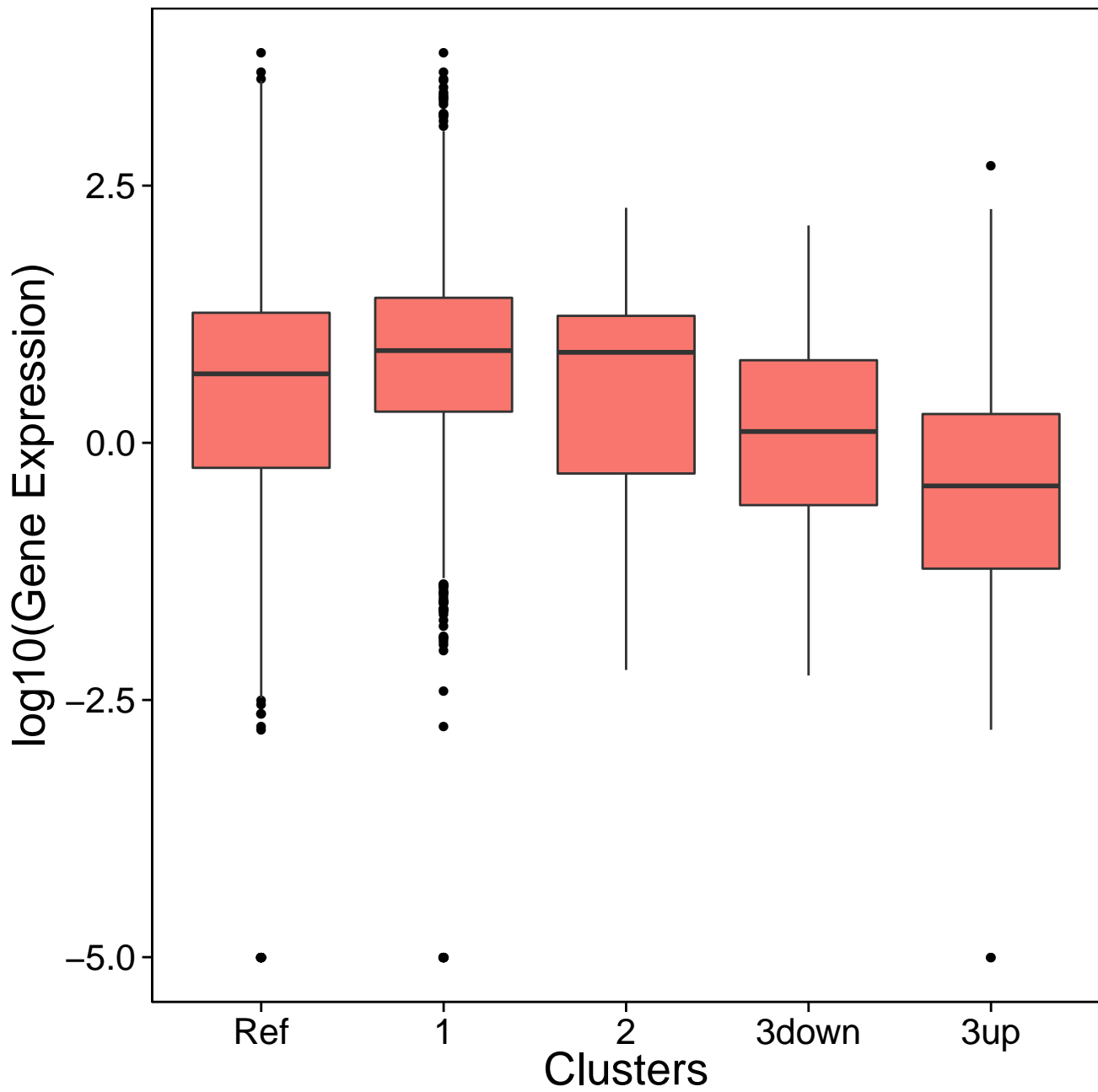

Supplement: Additional file 8 — Gene expression patterns for each CGI + SS clusters in colon tissues. (PDF 5.18 kb) [file 12864_2015_1994_MOESM8_ESM.pdf]

# Color Key

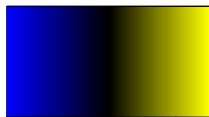

0.2 0.6  
Methylation

Cluster 3up  
Survival  
HER2  
ER

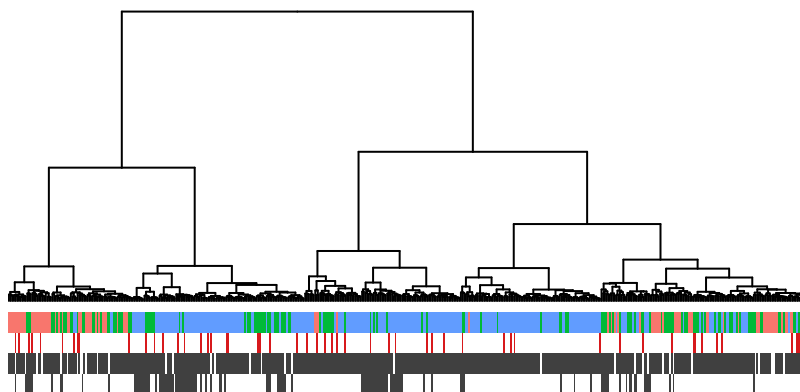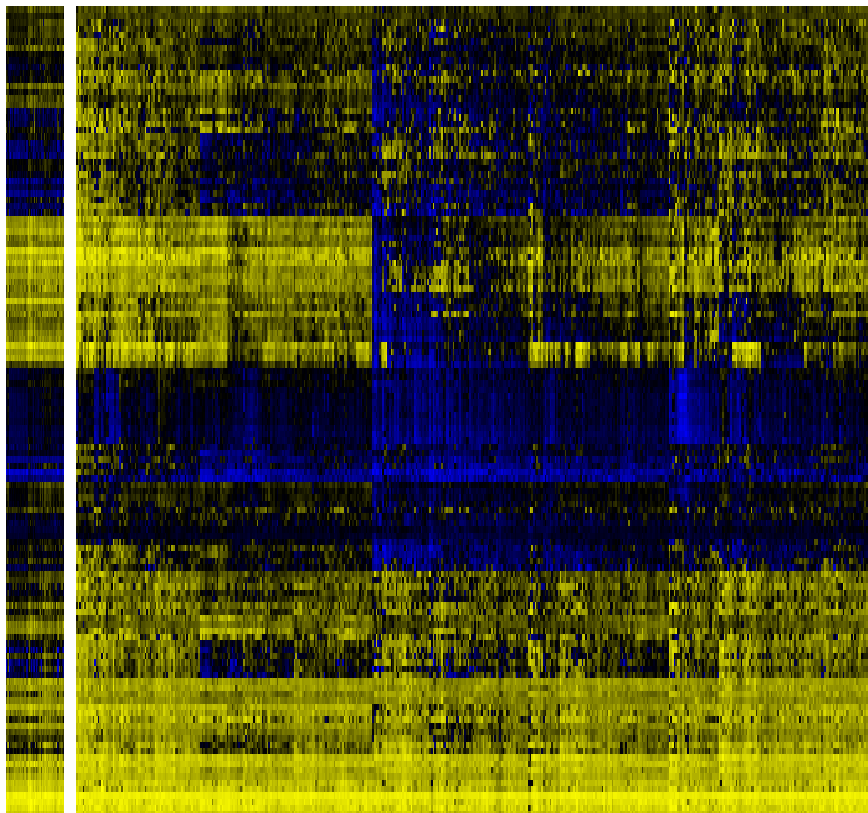

Supplement: Additional file 10 — Hierarchical clustering of breast cancer patients based on the average methylation level of CGI + SS associated with cluster 3down. (PDF 379 kb) [file 12864_2015_1994_MOESM10_ESM.pdf]

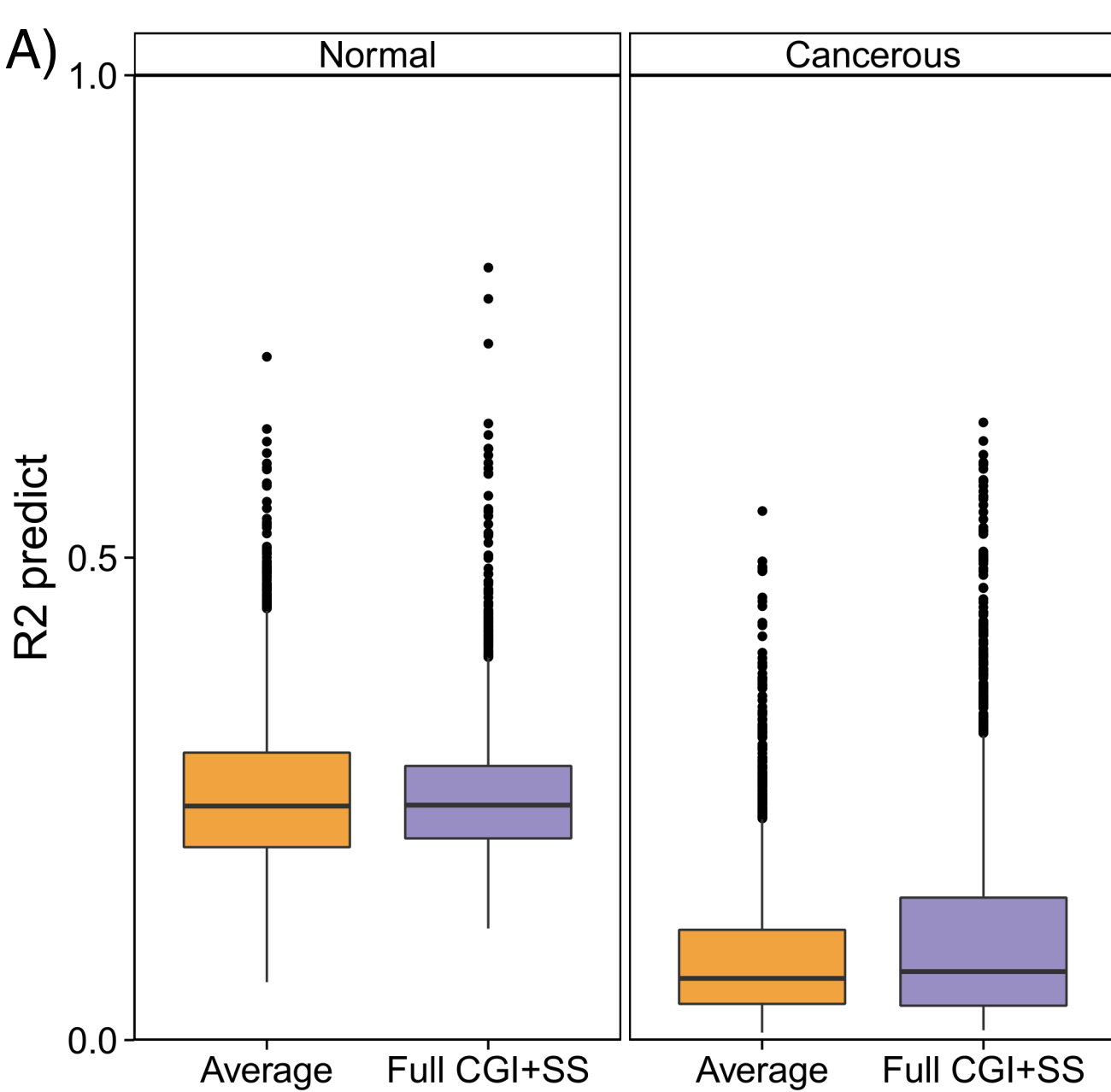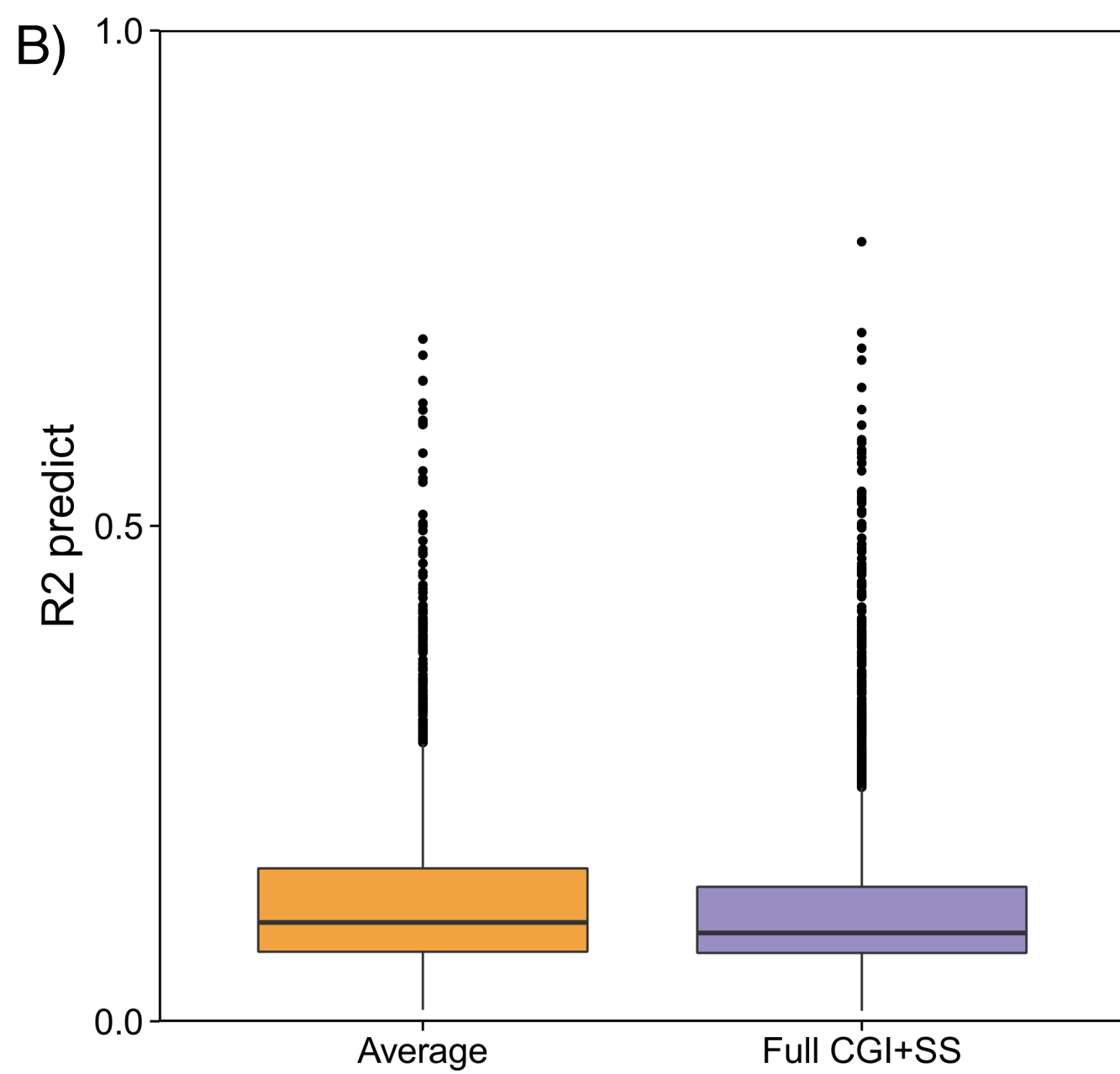

Supplement: Additional file 11 — Impact of DNA methylation on gene expression prediction. (PDF 156 kb) [file 12864_2015_1994_MOESM11_ESM.pdf]

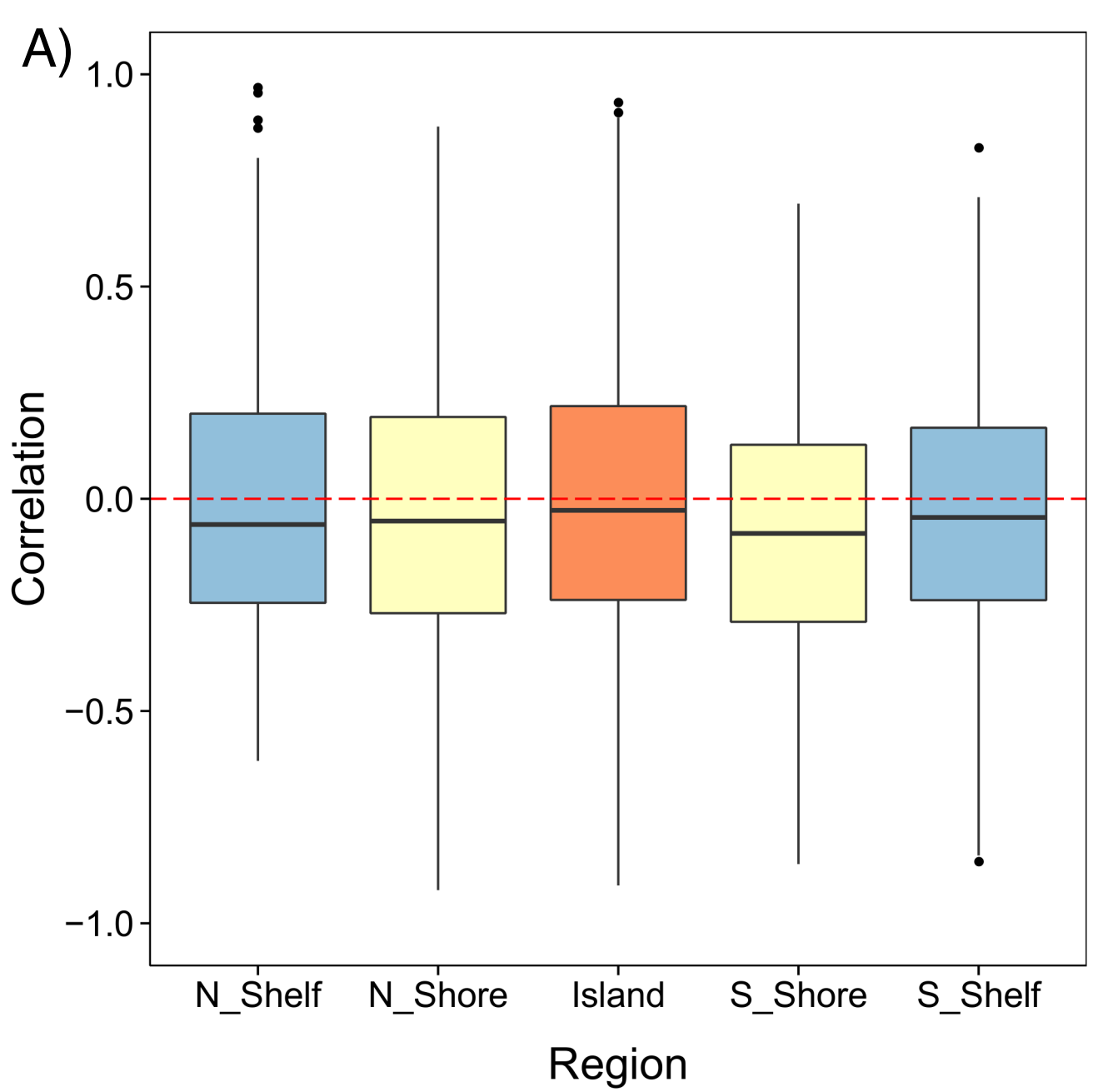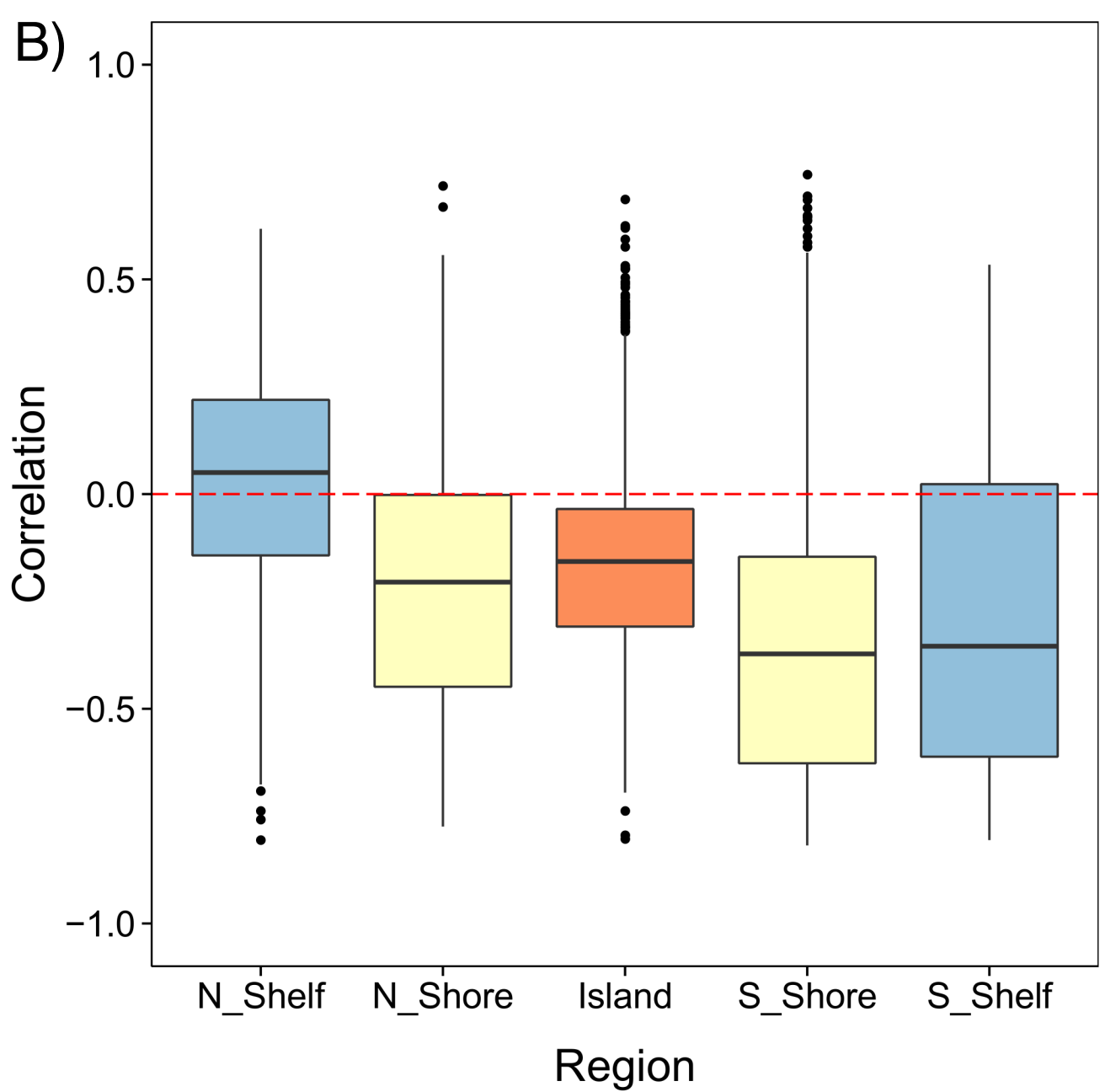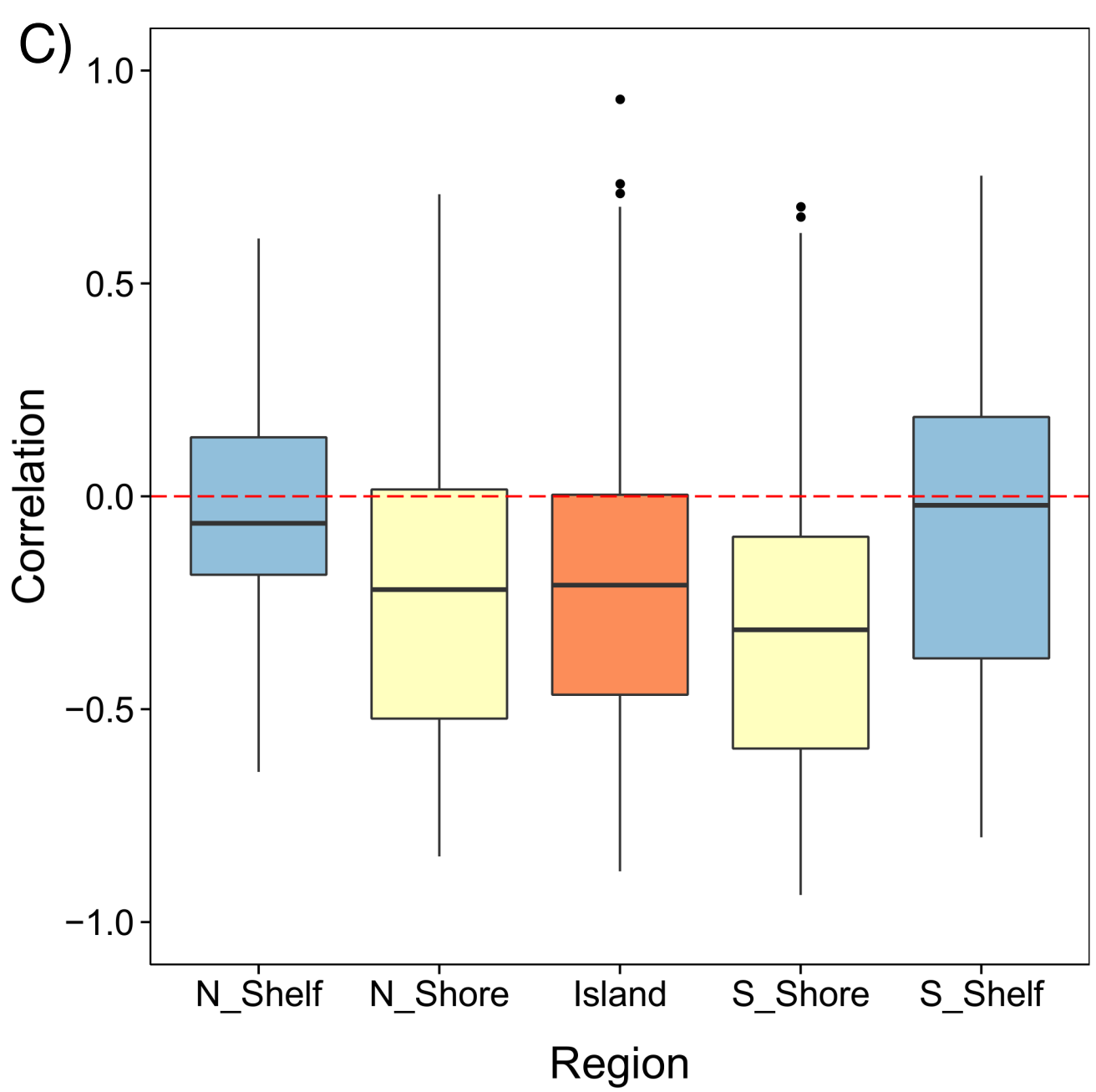

Supplement: Additional file 13 — Methylation association with gene expression by regions. Panel A. Colon cancerous samples. Panel B. Lung normal samples. Panel C. Lung cancerous samples. (PDF 252 kb) [file 12864_2015_1994_MOESM13_ESM.pdf]

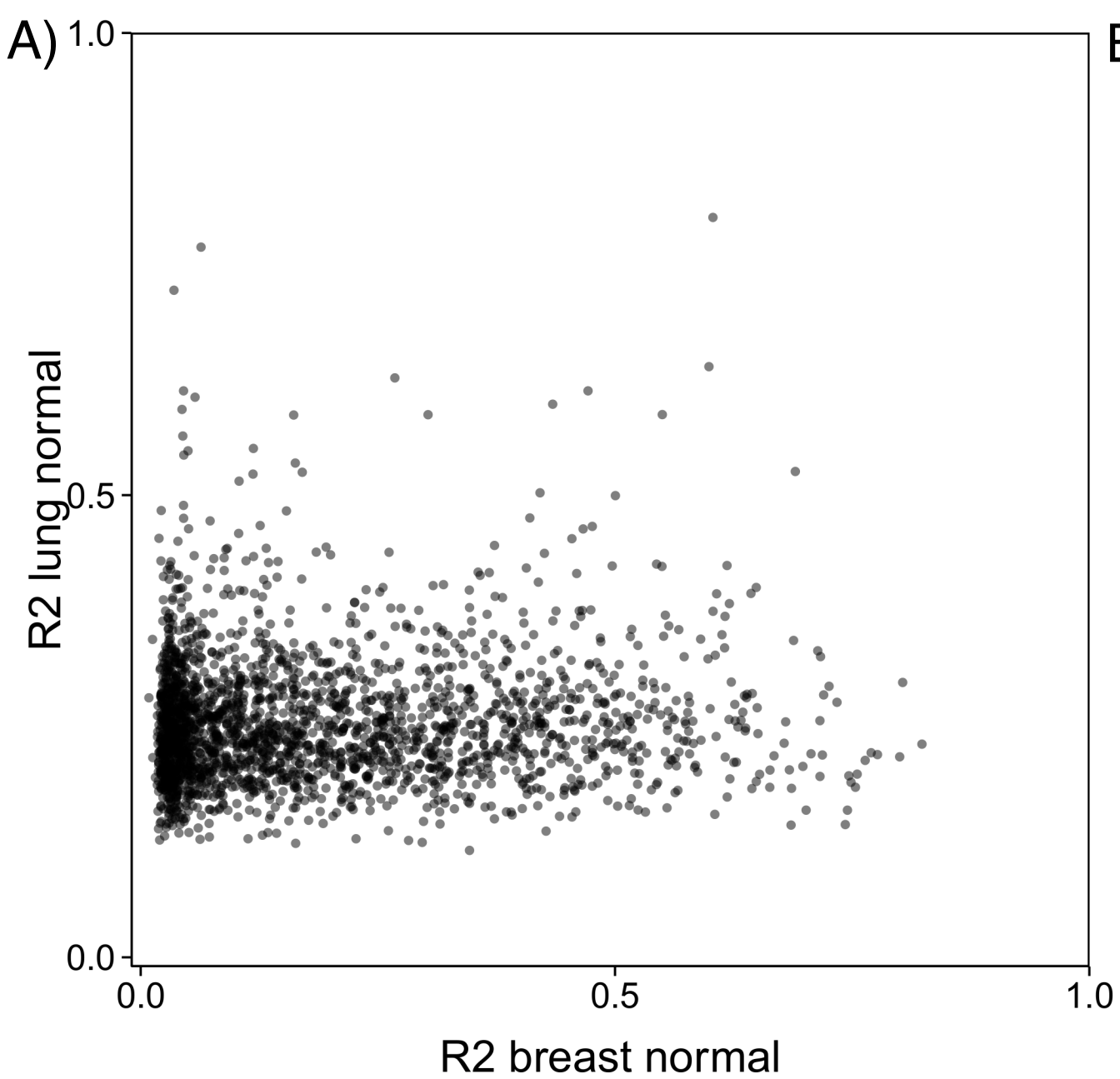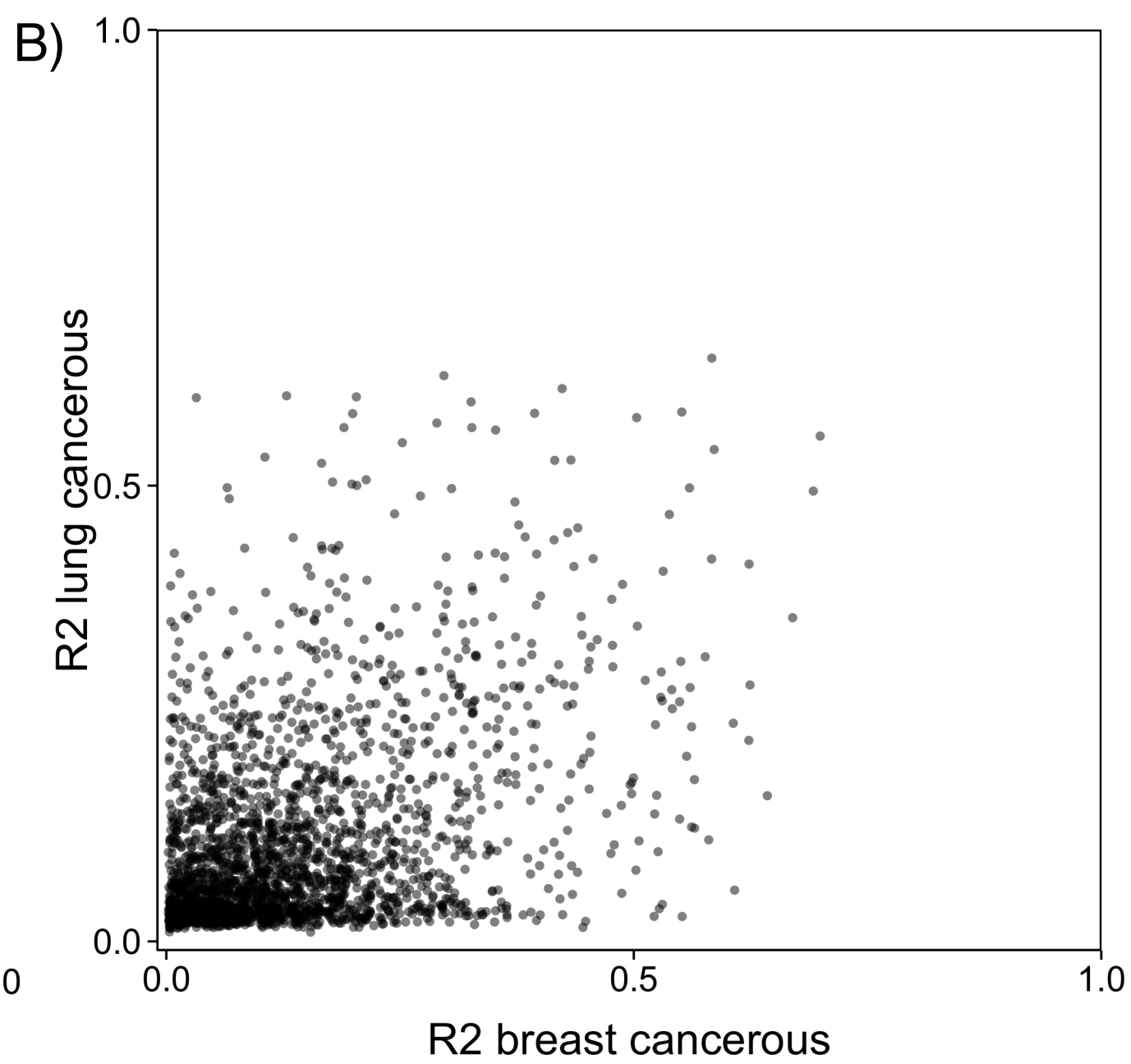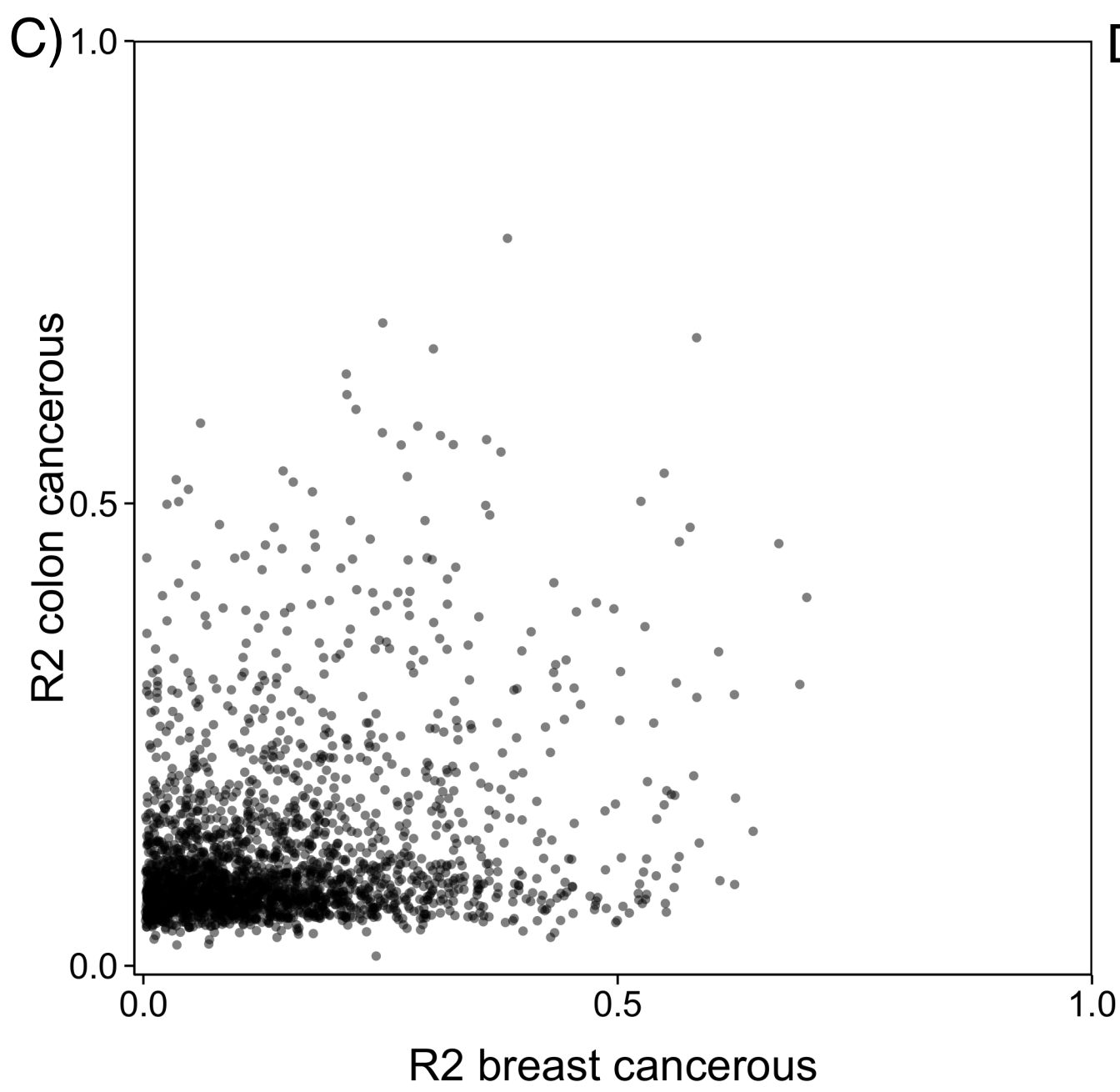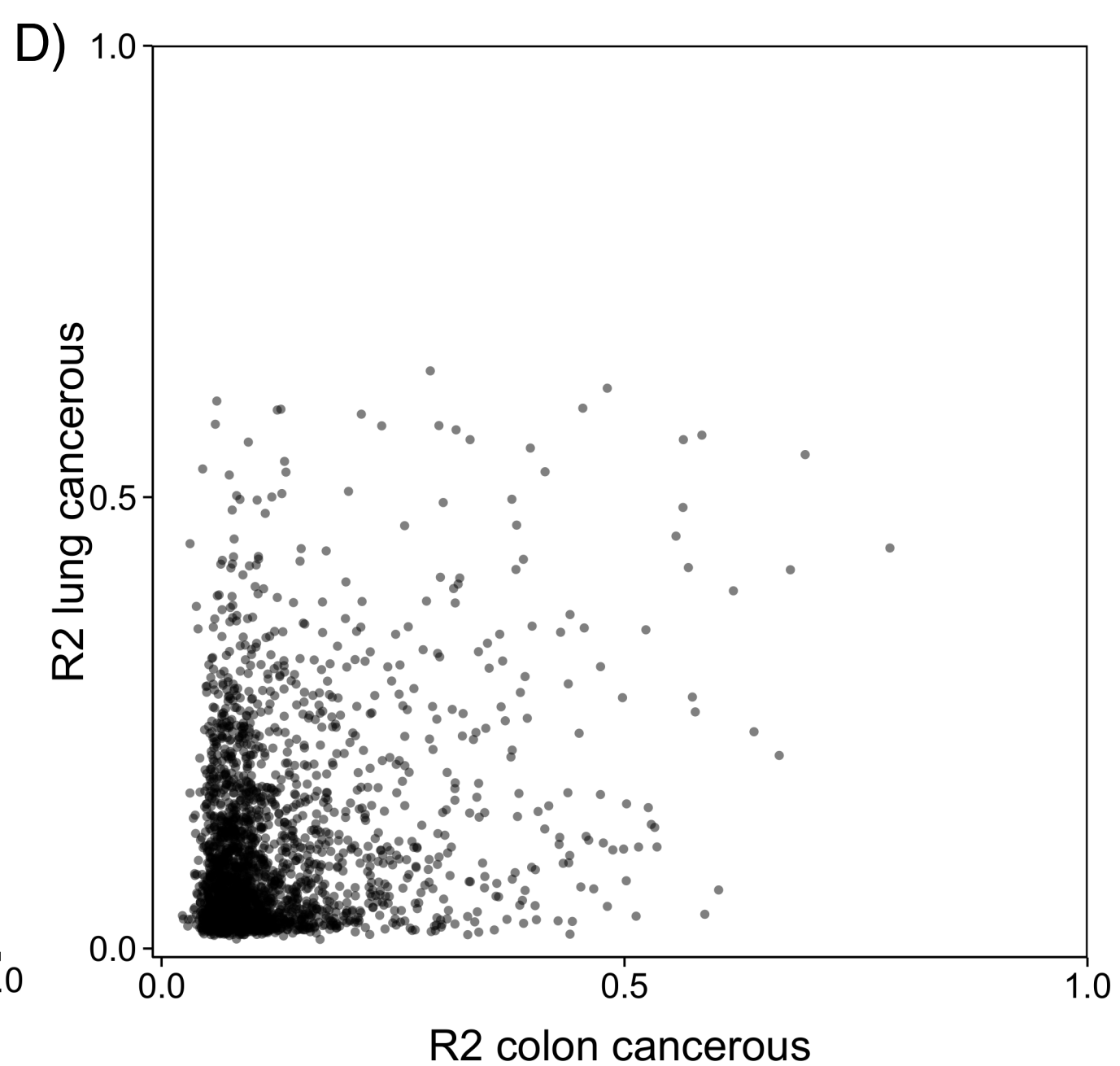

Supplement: Additional file 14 — Tissue-specificity of methylation for gene expression regulation in normal and cancerous tissues. Panel A. normal breast and lung samples. Panel B. cancerous breast and colon samples. Panel C. cancerous colon and lung samples. Panel D. cancerous lung and breast samples. (PDF 714 kb) [file 12864_2015_1994_MOESM14_ESM.pdf]

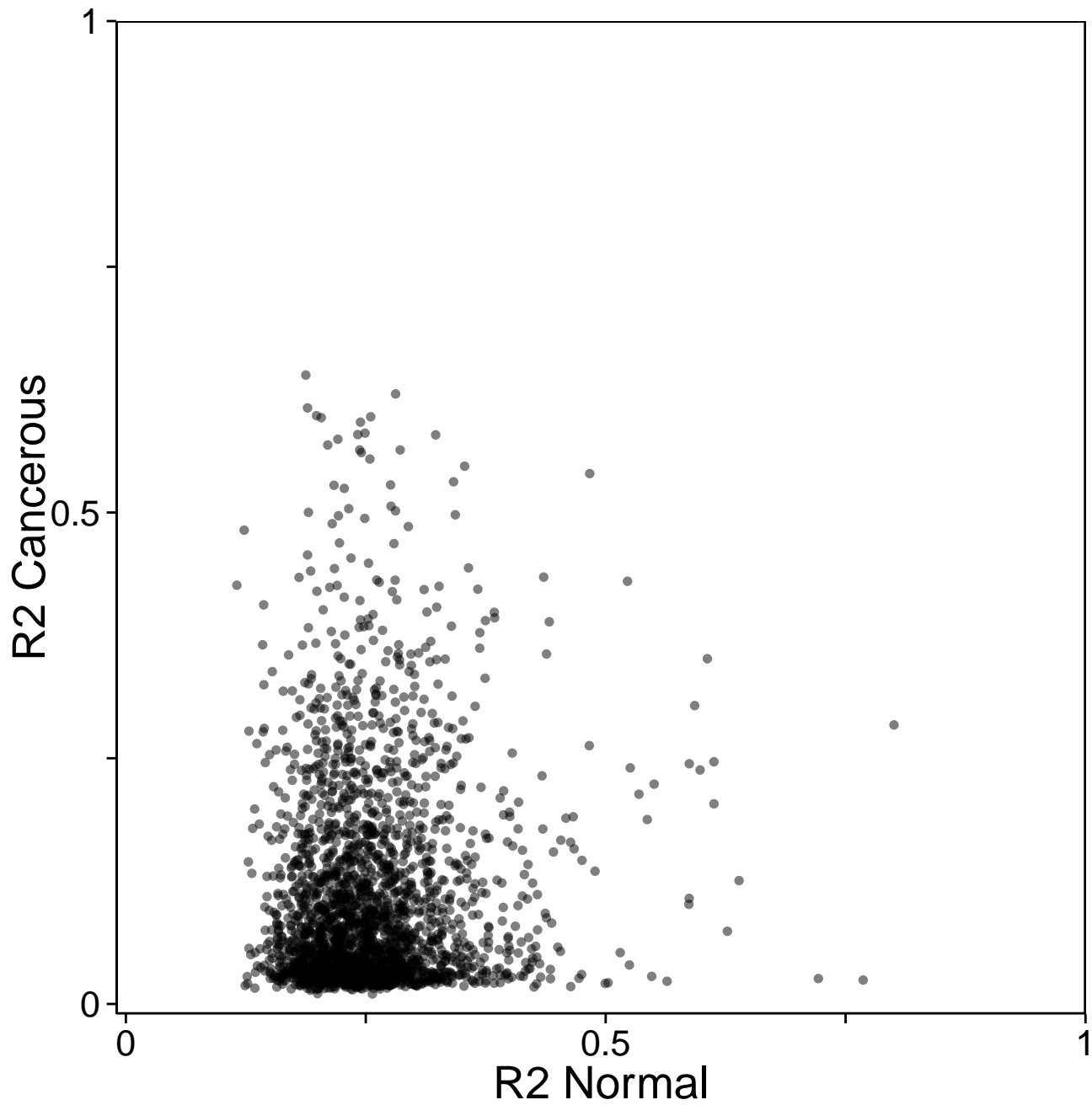

Supplement: Additional file 15 — Shift of epigenetic regulation between normal and cancerous lung tissues. (PDF 135 kb) [file 12864_2015_1994_MOESM15_ESM.pdf]

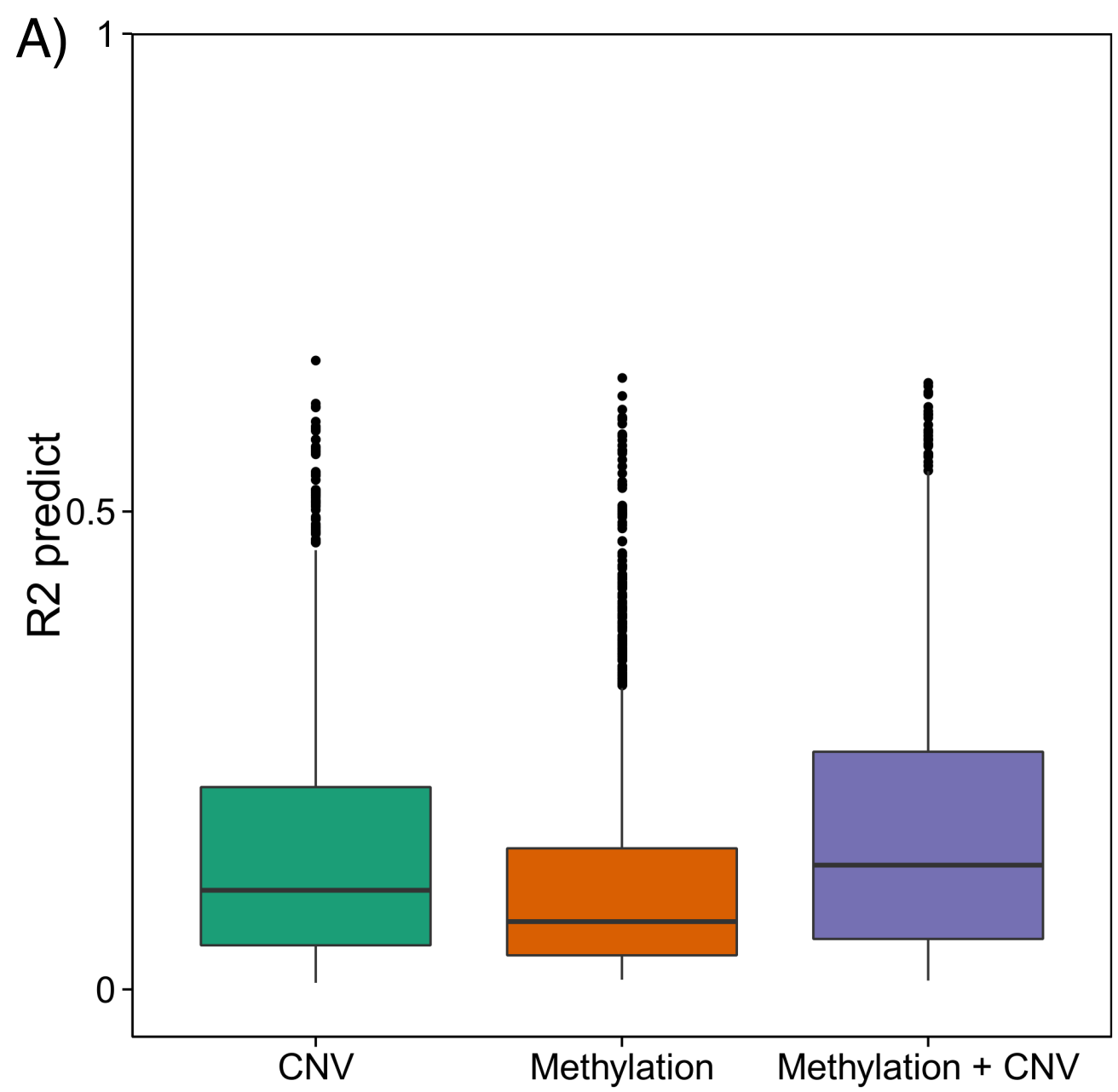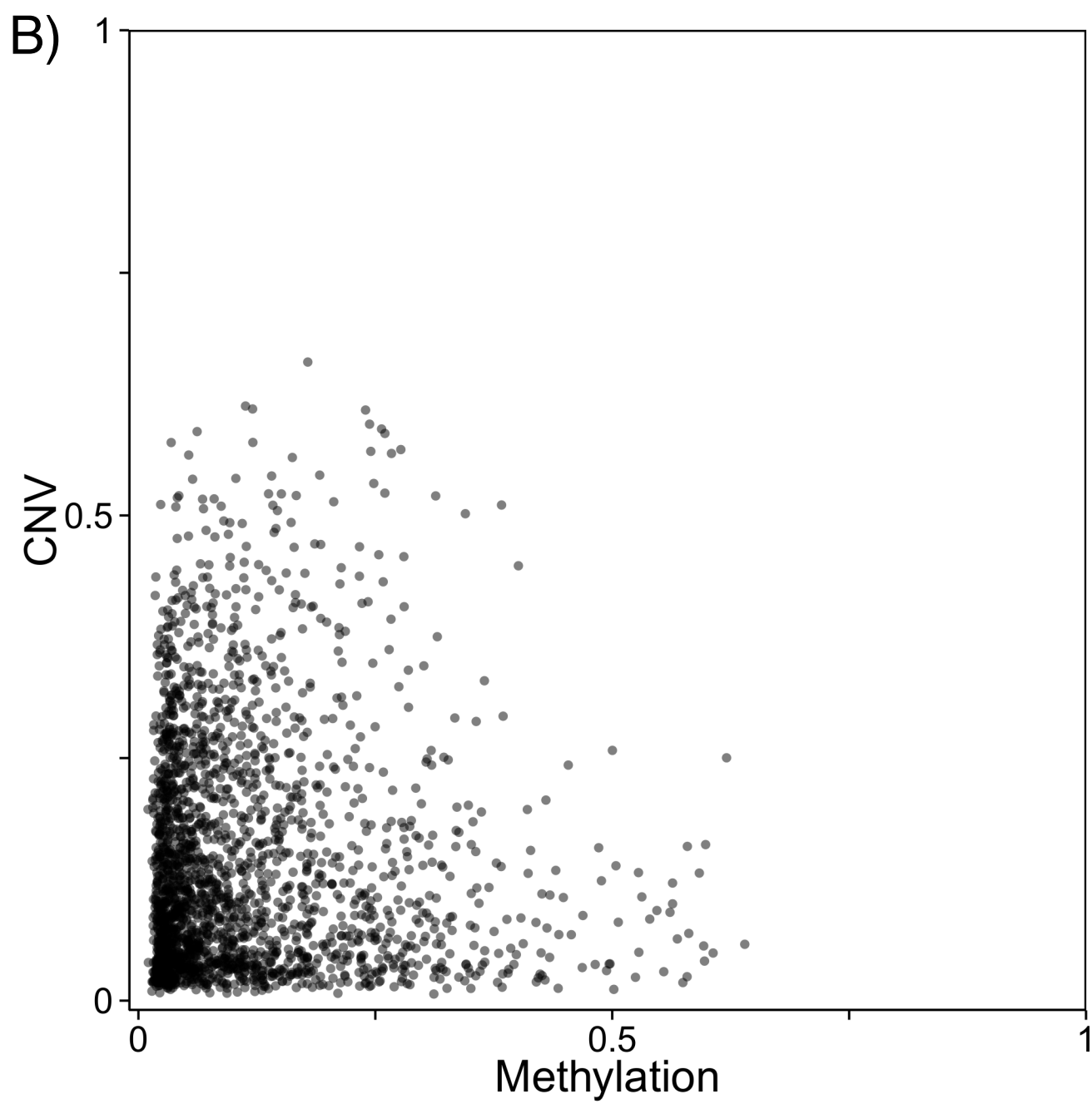

Supplement: Additional file 16 — Specific increase of predictive power for aberrant CNV by including copy number profiles in lung tissues. (PDF 415 kb) [file 12864_2015_1994_MOESM16_ESM.pdf]

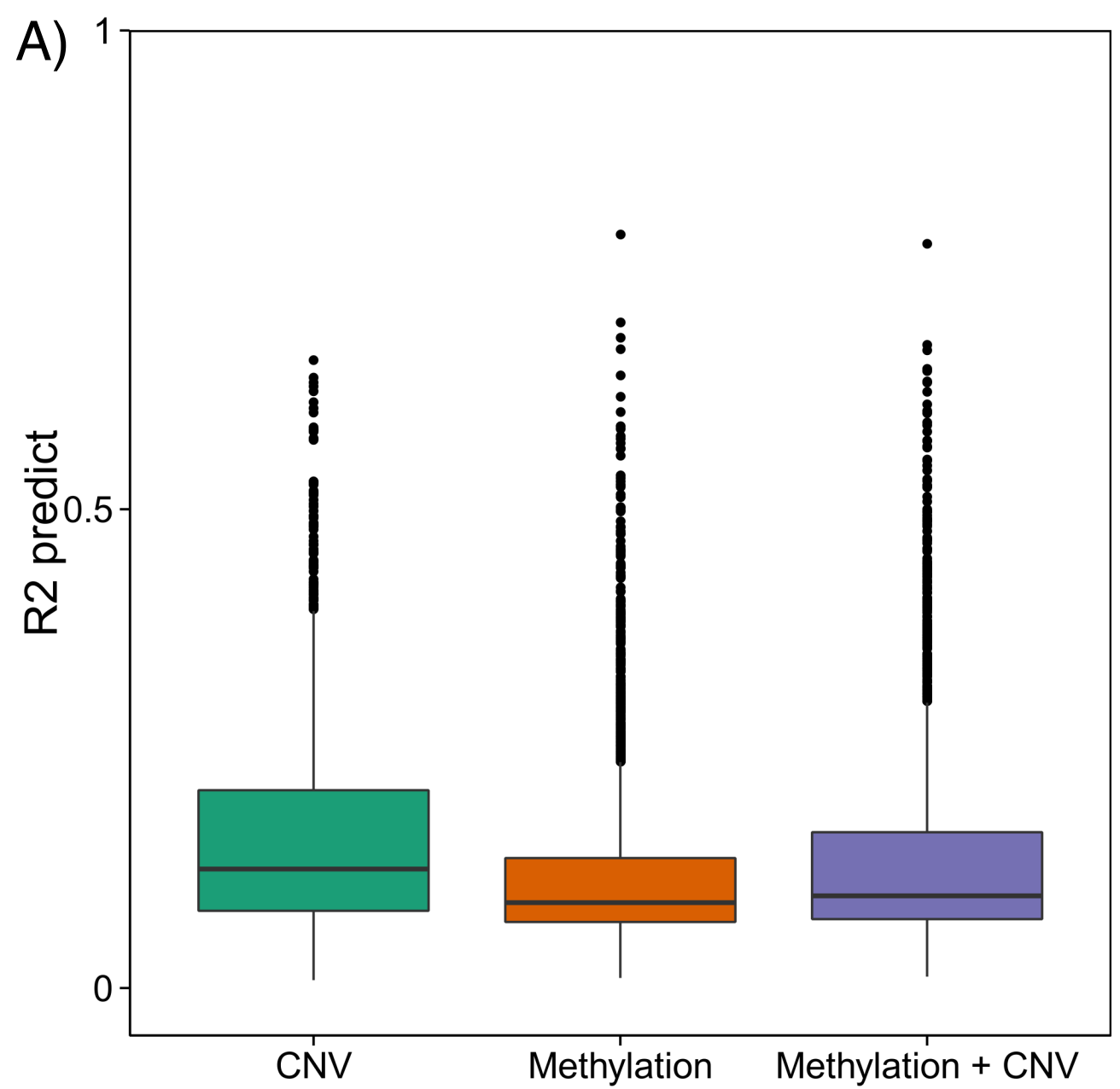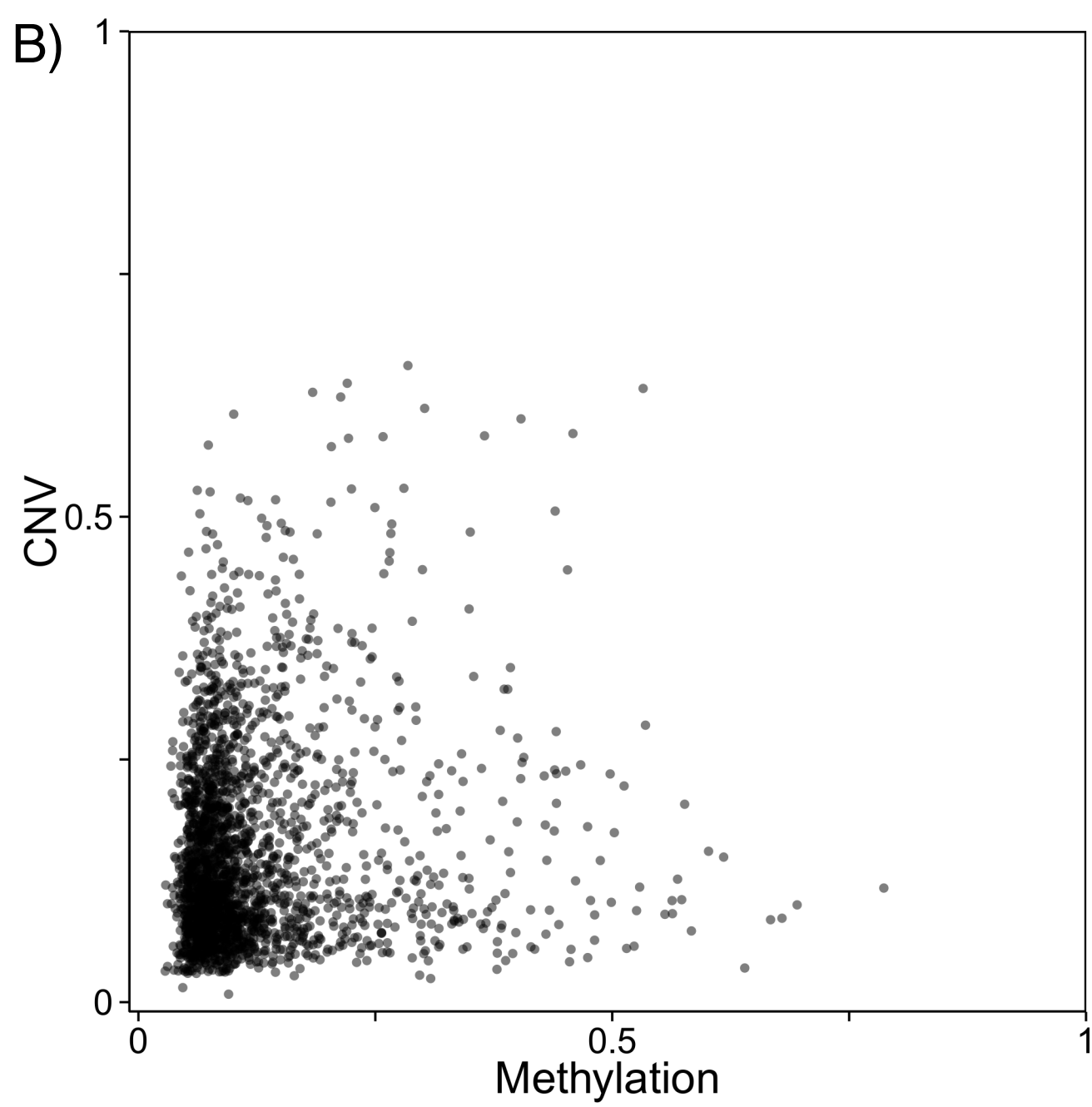

Supplement: Additional file 17 — Specific increase of predictive power for aberrant CNV by including copy number profiles in colon tissues. (PDF 367 kb) [file 12864_2015_1994_MOESM17_ESM.pdf]
