# Supplementary material for: Changes in correlation between promoter methylation and gene expression in cancer
Source: BMC Genomics. 2015 Oct 28;16:873. doi: 10.1186/s12864-015-1994-2 (PMC4625954; doi:10.1186/s12864-015-1994-2)
Supplement: Additional file 6 — Gene ontology analysis for the genes associated with cancer-specific cluster 3. (PDF 72 kb) [file 12864_2015_1994_MOESM6_ESM.pdf]

Table 1: Gene Ontology Analysis given the cluster assignment for cancerous breast tissues. **Cluster**: Cluster assignment of a gene the CGI+SS methylation pattern . **Description**: Description of the biological processes enriched (ranked by p-value). **p-value**: Fisher's exact test p-value to assess whether the cluster is enriched in the given biological process compared to the other remaining clusters.

Cluster	Description	p-value
1	metabolic process	8.85×10^{-168}
1	organic substance metabolic process	5.31×10^{-156}
1	cellular metabolic process	5.56×10^{-151}
1	primary metabolic process	7.47×10^{-150}
1	response to stimulus	5.91×10^{-103}
1	cellular response to stimulus	1.64×10^{-79}
1	cellular component organization or biogenesis	8.00×10^{-79}
1	cellular component organization	6.17×10^{-77}
1	single-organism metabolic process	3.35×10^{-67}
1	localization	1.04×10^{-66}
2	**No significant enrichment**	
3down	**No significant enrichment**	
3up	multicellular organismal process	1.89×10^{-212}
3up	single-multicellular organism process	7.13×10^{-209}
3up	developmental process	2.71×10^{-198}
3up	single-organism developmental process	3.94×10^{-197}
3up	multicellular organismal development	2.32×10^{-191}
3up	anatomical structure development	5.25×10^{-180}
3up	system development	6.83×10^{-169}
3up	organic cyclic compound biosynthetic process	1.13×10^{-131}
3up	aromatic compound biosynthetic process	1.21×10^{-130}
3up	cellular nitrogen compound biosynthetic process	1.21×10^{-130}

Table 2: Gene Ontology Analysis given the cluster assignment for lung cancerous tissues. **Cluster**: Cluster assignment of a gene the CGI+SS methylation pattern. **Description**: Description of the biological processes enriched (ranked by p-value). **p-value**: Fisher's exact test p-value to assess whether the cluster is enriched in the given biological process compared to the other remaining clusters.

Cluster	Description	p-value
1	single-organism metabolic process	1.79×10^{-76}
1	protein metabolic process	7.20×10^{-70}
1	cellular protein metabolic process	6.70×10^{-62}
1	establishment of localization	5.80×10^{-56}
1	transport	2.59×10^{-54}
1	response to stress	1.39×10^{-51}
1	macromolecule modification	2.89×10^{-47}
1	phosphorus metabolic process	4.36×10^{-47}
1	phosphate-containing compound metabolic process	1.50×10^{-46}
1	cellular protein modification process	2.63×10^{-45}
2	**No significant enrichment**	
3down	**No significant enrichment**	
3up	multicellular organismal process	3.51×10^{-241}
3up	single-multicellular organism process	4.35×10^{-235}
3up	developmental process	3.16×10^{-216}
3up	single-organism developmental process	2.64×10^{-213}
3up	multicellular organismal development	1.24×10^{-204}
3up	anatomical structure development	5.60×10^{-198}
3up	system development	6.78×10^{-185}
3up	macromolecule biosynthetic process	3.66×10^{-155}
3up	cellular developmental process	2.77×10^{-154}
3up	cellular differentiation	2.08×10^{-153}

Table 3: Gene Ontology Analysis given the cluster assignment for colon cancerous tissues. **Cluster**: Cluster assignment of a gene the CGI+SS methylation pattern . **Description**: Description of the biological processes enriched (ranked by p-value). **p-value**: Fisher's exact test p-value to assess whether the cluster is enriched in the given biological process compared to the other remaining clusters.

Cluster	Description	p-value
1	metabolic process	8.79×10^{-198}
1	organic substance metabolic process	5.46×10^{-185}
1	cellular metabolic process	5.01×10^{-181}
1	primary metabolic process	8.73×10^{-179}
1	macromolecule metabolic process	1.71×10^{-149}
1	cellular macromolecule metabolic process	1.73×10^{-139}
1	nitrogen compound metabolic process	2.19×10^{-120}
1	cellular nitrogen compound metabolic process	2.49×10^{-115}
1	organic cyclic compound metabolic process	9.72×10^{-114}
1	cellular aromatic compound metabolic process	2.35×10^{-110}
2	**No significant enrichment**	
3down	**No significant enrichment**	
3up	multicellular organismal process	1.83×10^{-246}
3up	single-multicellular organism process	6.68×10^{-242}
3up	single-organism developmental process	2.20×10^{-221}
3up	developmental process	2.20×10^{-221}
3up	multicellular organismal development	5.86×10^{-211}
3up	anatomical structure development	4.94×10^{-199}
3up	system development	2.42×10^{-187}
3up	cellular developmental process	4.81×10^{-145}
3up	cell differentiation	2.83×10^{-144}
3up	organ development	8.20×10^{-130}
