# Supplementary material for: Changes in correlation between promoter methylation and gene expression in cancer
Source: BMC Genomics. 2015 Oct 28;16:873. doi: 10.1186/s12864-015-1994-2 (PMC4625954; doi:10.1186/s12864-015-1994-2)
Supplement: Additional file 7 — Gene expression patterns for each CGI + SS clusters in lung tissues. (PDF 286 kb) [file 12864_2015_1994_MOESM7_ESM.pdf]

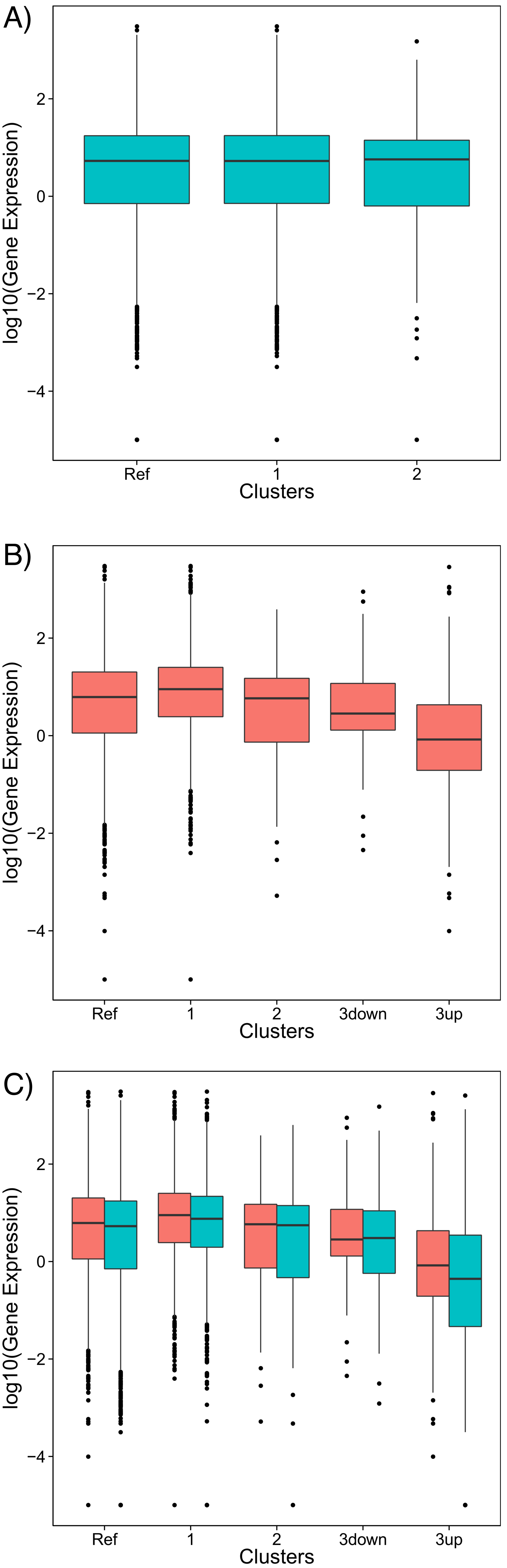
A)
B)
C)
log10(Gene Expression)
2
0
−2
−4
Ref
1
2
3down
3up
Clusters
